# Mild Deficits in Fear Learning: Evidence from Humans and Mice with Cerebellar Cortical Degeneration

**DOI:** 10.1523/ENEURO.0365-23.2023

**Published:** 2024-02-22

**Authors:** Giorgi Batsikadze, Johanna Pakusch, Michael Klein, Thomas Michael Ernst, Andreas Thieme, Seyed Ali Nicksirat, Katharina Marie Steiner, Enzo Nio, Erhan Genc, Stefan Maderwald, Cornelius Deuschl, Christian Josef Merz, Harald H. Quick, Melanie D. Mark, Dagmar Timmann

**Affiliations:** ^1^Department of Neurology and Center for Translational Neuro- and Behavioral Sciences (C-TNBS), Essen University Hospital, University of Duisburg-Essen, 45147 Essen, Germany; ^2^Erwin L. Hahn Institute for Magnetic Resonance Imaging, University of Duisburg-Essen, 45141 Essen, Germany; ^3^Behavioral Neuroscience, Ruhr University Bochum, 44801 Bochum, Germany; ^4^LVR-Hospital Essen, Department of Psychiatry and Psychotherapy, Medical Faculty, University of Duisburg-Essen, 45147 Essen, Germany; ^5^Department of Psychology and Neurosciences, Leibniz Research Centre for Working Environment and Human Factors, Technical University of Dortmund (IfADo), 44139 Dortmund, Germany; ^6^Institute of Diagnostic and Interventional Radiology and Neuroradiology and C-TNBS, Essen University Hospital, University of Duisburg-Essen, 45147 Essen, Germany; ^7^Department of Cognitive Psychology, Institute of Cognitive Neuroscience, Ruhr University Bochum, 44801 Bochum, Germany; ^8^High-Field and Hybrid MR Imaging, Essen University Hospital, University of Duisburg-Essen, 45147 Essen, Germany

**Keywords:** associative learning, cerebellar atrophy, cerebellum, fear conditioning, human, mouse model

## Abstract

Functional brain imaging studies in humans suggest involvement of the cerebellum in fear conditioning but do not allow conclusions about the functional significance. The main aim of the present study was to examine whether patients with cerebellar degeneration show impaired fear conditioning and whether this is accompanied by alterations in cerebellar cortical activations. To this end, a 2 d differential fear conditioning study was conducted in 20 cerebellar patients and 21 control subjects using a 7 tesla (7 T) MRI system. Fear acquisition and extinction training were performed on day 1, followed by recall on day 2. Cerebellar patients learned to differentiate between the CS+ and CS−. Acquisition and consolidation of learned fear, however, was slowed. Additionally, extinction learning appeared to be delayed. The fMRI signal was reduced in relation to the prediction of the aversive stimulus and altered in relation to its unexpected omission. Similarly, mice with cerebellar cortical degeneration (spinocerebellar ataxia type 6, SCA6) were able to learn the fear association, but retrieval of fear memory was reduced. In sum, cerebellar cortical degeneration led to mild abnormalities in the acquisition of learned fear responses in both humans and mice, particularly manifesting postacquisition training. Future research is warranted to investigate the basis of altered fMRI signals related to fear learning.

## Significance Statement

Humans and mice with pure cerebellar cortical degeneration showed deficits in the acquisition of learned fear, but abnormalities were mild. Given that cerebellar fMRI signals predominantly reflect mossy fiber input, changes in cerebellar activations suggest that input signals related to the prediction and unexpected omission of the aversive US are altered in patients with cerebellar cortical degeneration. Importantly, these differences cannot be explained by cerebellar atrophy because cerebellar fMRI signal related to the presentation of the aversive stimulus (US) was not significantly different from controls. Future research is warranted to study the exact nature of cerebellar input signals related to fear learning.

## Introduction

Even though involvement of the cerebellum in the control of emotions has been known for a long time, it has only recently gained increasing interest (Schutter, 2020; Adamaszek et al., 2022). Most studies have examined the contribution of the cerebellum to the regulation of fear, an important emotion for survival (Timmann et al., 2010, for review; Mark et al., 2022). Lesions in the vermis and cerebellar nuclei have been found to impact defense behaviors in rodents (Supple et al., 1988; Sacchetti et al., 2002). In addition, the cerebellum has been shown to contribute not only to innate affective and defensive behavior but also to learned fear responses. For instance, lesions in the vermis severely reduce fear-conditioned bradycardia in rodents (Lavond et al., 1984; Supple and Leaton, 1990). The cerebellar vermis also contributes to fear-conditioned freezing (Sacchetti et al., 2002; Watson et al., 2013). Likewise, the cerebellum has multiple anatomical connections with the neural fear network including the periaqueductal gray, amygdala, the anterior cingulate, and pre- and infralimbic cortex (Middleton and Strick, 2001; Koutsikou et al., 2014; Watson et al., 2014; Apps and Strata, 2015, for review; Farley et al., 2016; Badura et al., 2018; Frontera et al., 2020; Pisano et al., 2021; Jung et al., 2022).

Functional brain imaging studies have supported the involvement of the cerebellum in fear conditioning in humans (Lange et al., 2015). Cerebellar activations linked to the prediction of learned threat go beyond the vermis and include the cerebellar hemispheres (Ploghaus et al., 1999; Fischer et al., 2000; Frings et al., 2002; Ernst et al., 2019). Furthermore, Ploghaus et al. (1999) and Ernst et al. (2019) observed prominent cerebellar activations during the unexpected omission of an expected aversive stimulus suggesting that the human cerebellum contributes to the processing of prediction errors which drive fear extinction learning (Doubliez et al., 2023).

However, despite these findings, functional brain imaging studies do not distinguish whether an activated brain region is necessary for a given brain function (Karnath et al., 2019). Studies in patients with brain lesions help to answer this question. To date, only two studies have examined patients to assess the cerebellar contribution to fear learning (Maschke et al., 2000, 2002). Maschke et al. (2002) tested single-cue fear conditioning in a small group of five patients with surgical cerebellar lesions. Controls, but not cerebellar patients, showed a significant learning-related decrease of heart rate toward the conditioned stimulus (CS) in unpaired fear recall trials. The incidence of skin conductance responses (SCRs) showed no significant learning-related increase, neither in patients nor in controls. In another study, fear conditioning was assessed as part of a fear-potentiated startle paradigm in a group of 10 patients with predominantly surgical lesions (Maschke et al., 2000). Both patients and controls showed no significant fear learning-related changes of heart rate or SCR incidences. The short (2 s) CS/unconditioned stimulus (US) time window, however, limited the assessment of autonomic fear responses related to the CS unbiased by the US.

The main aim of the present study was to investigate potential alterations in the acquisition and extinction of learned fear responses in a larger group of patients with cerebellar cortical degeneration. A secondary aim was to assess whether cerebellar cortical activations related to the prediction and unexpected omission of the aversive stimuli are altered in cerebellar patients. We hypothesized that cerebellar cortical activations were reduced in patients. To this end, cerebellar patients and matched controls performed a fear conditioning paradigm in a 7 T MRI scanner. For comparative analysis, we investigated fear conditioning in a cerebellar cortical degenerative mouse model known as spinocerebellar ataxia type 6 (SCA6). Our SCA6 mouse model, CT-longQ27^PC^, is characterized by the expression of the P/Q-type calcium channel carboxy-terminus (CT) containing 27 polyglutamine repeats in specifically Purkinje cells, the same number of polyglutamine repeats found in the human disease for SCA6. Overexpression of the long CT fragment in Purkinje cells results in Purkinje cell loss starting at 4 months of age which is augmented with age and ataxia starting at 8 months of age (Mark et al., 2015). Moreover, CT-longQ27^PC^ mice display an increase in Purkinje cell aggregates during late stages (13–16 months of age) of the disease (Bohne et al., 2022).

## Materials and Methods

### Human study

#### Participants

A total of 23 cerebellar patients (12 males and 11 females; mean age, 56.1 ± 11.1 years; range, 24–70 years) and 25 age- and sex-matched healthy controls (15 males and 10 females; mean age, 57 ± 11.1 years; range, 24–77 years) performed the experiment. One participant had to be excluded due to abnormal findings on neurological examination, two due to technical errors (i.e., volume orientation mix-ups or loss of adjustment volume settings), and another five due to motion and related image artifacts. Thus, 20 cerebellar patients (10 males and 10 females; mean age, 55.7 ± 11.8 years; range, 24–70 years) and 21 controls (13 males and 8 females; mean age, 56.8 ± 11.8 years; range, 24–77 years) were included in the final data analysis. All patients suffered from a pure form of cerebellar cortical degeneration. Of the 20 cerebellar patients, 14 presented with hereditary ataxia [SCA6 ( *n* = 6), SCA8 (*n* = 2), SCA14 (*n* = 1), or autosomal dominant cerebellar ataxia type 3 (ADCA III, *n* = 5)], and six with sporadic adult-onset ataxia of unknown etiology (SAOA; Table 1). Nineteen cerebellar patients and 18 controls were right-handed, one patient and one control were left-handed, and two controls were ambidextrous based on the Edinburgh Handedness Inventory (Oldfield, 1971).

**Table 1.** Basic characteristics of cerebellar patients

Patient	Diagnosis	Age (years)	Sex	Duration (years)	SARA	ICARS
1	SCA14	58	M	17	21	33
2	ADCA III	55	F	22	17	30
3	ADCA III	25	M	2	6	9
4	SAOA	70	M	15	22	48
5	ADCA III	56	M	19	24	44
6	SCA6	58	F	8	19	46
7	SAOA	65	M	17	17	30
8	ADCA III	30	F	1	7	16
9	SCA8	60	M	9	15	23
10	SAOA	67	F	14	16	31
11	SAOA	55	F	5	19	22
12	SCA6	62	M	7	17	28
13	ADCA III	56	F	15	12	19
14	SAOA	66	M	2	18	26
15	SCA6	52	M	15	6	15
16	SAOA	59	F	9	12	28
17	SCA6	39	F	1	12	21
18	SCA8	62	F	12	4	5
19	SCA6	53	F	Presymptomatic	1	3
20	SCA6	65	M	27	15	30

Listed are age and disease duration at examination; SCA6, SCA8, SCA14, spinocerebellar ataxia types 6, 8, and 14; ADCA III, autosomal dominant cerebellar ataxia type III; SAOA, sporadic adult-onset ataxia of unknown etiology. Severity of ataxia was rated using the ICARS and the SARA. Total SARA and ICARS scores are given. Maximum ICARS score is 100; maximum SARA score is 40 (Trouillas et al., 1997; Schmitz-Hübsch et al., 2006).

Every participant received instructions to abstain from consuming alcohol for a minimum of 24 h before the experiment. The study was approved by the Ethics Committee of the University Hospital Essen (proposal ID 16-7255-BO) and conforms to the principles laid down in the Declaration of Helsinki. All participants provided informed consent and received compensation of 80 Euros for their participation.

The severity of cerebellar symptoms in cerebellar participants were assessed by three experienced neurologists (AT, SAN, KMS) based on the Scale for the Assessment and Rating of Ataxia (SARA; Schmitz-Hübsch et al., 2006) and the International Cooperative Ataxia Rating Scale (ICARS; Trouillas et al., 1997). We decided to perform SARA and ICARS, because the former is most commonly used in ataxia research and the latter includes a rating of disordered eye movement related to cerebellar dysfunction which is not included in SARA.

#### Depression-Anxiety-Stress-Scale-21

The Depression-Anxiety-Stress-Scale-21 (DASS-21, Henry and Crawford, 2005) questionnaire was used to assess participants’ depression, anxiety, and stress levels. The DASS-21 is a 21-question self-report with seven questions for each of the three subscales. On the depression subscale, a score of 0–9 is within the normal range, on the anxiety subscale a score of 0–7, and on the stress subscale a score of 0–14 (Lovibond et al., 1995).

#### Fear conditioning paradigm

**Figure 1.**
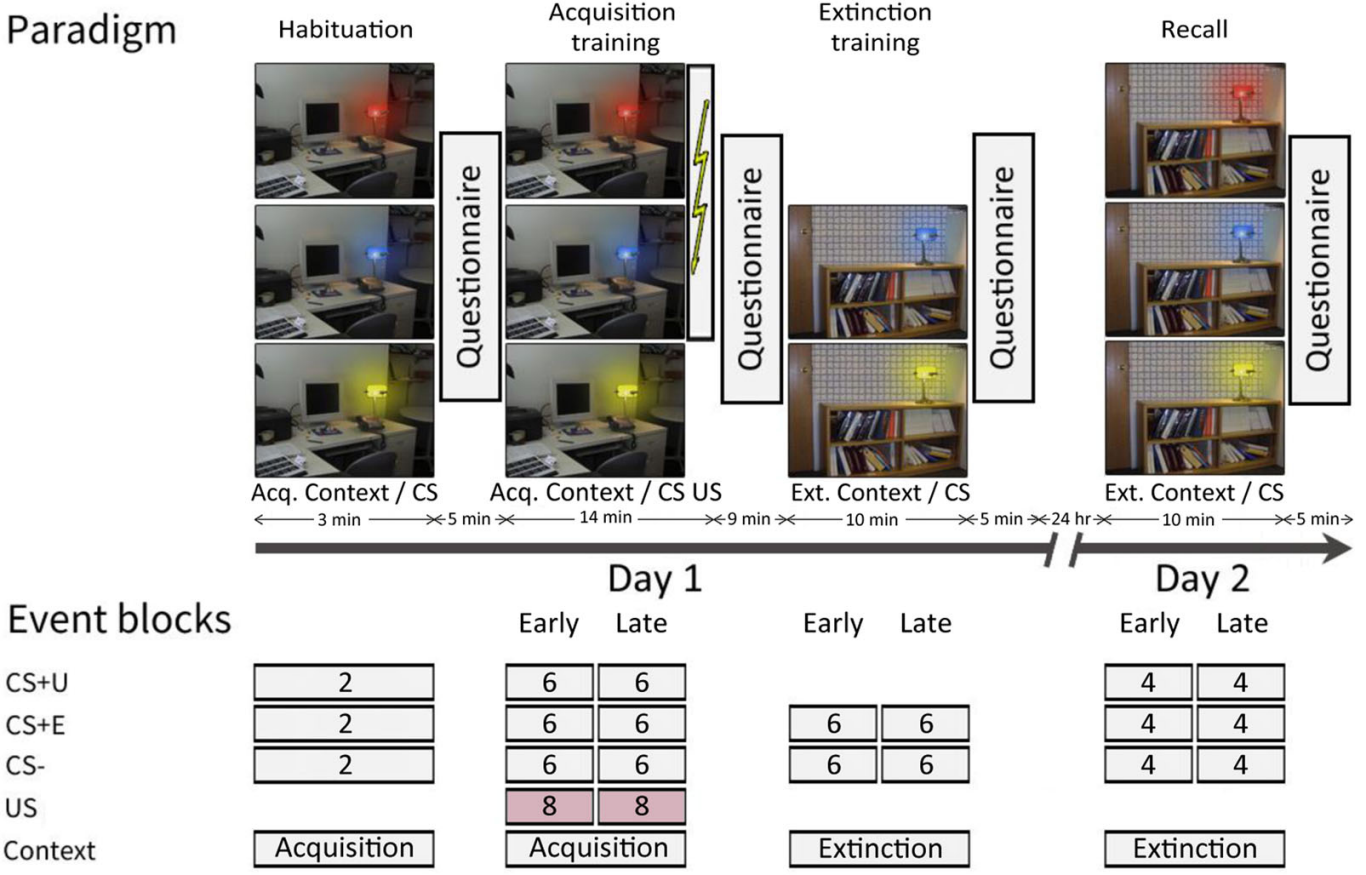
Experimental paradigm and event blocking scheme in the human study. Habituation and fear acquisition training were performed in the acquisition (Acq.) context. Extinction training and recall were performed in the extinction (Ext.) context. Contexts were represented by a photo of different office rooms either showing a desk (“office”) or a bookshelf (“library”). The conditioning stimuli (CSs) were represented by the same desk lamp, which emitted light in either blue, red, or yellow colors. The experimental paradigm closely followed the one described in Albayrak et al. (2023), which was based on the earlier study conducted by Milad et al. (2007).

The experiment was performed on 2 consecutive days. Figure 1 displays the experimental paradigm. Habituation, fear acquisition, and extinction training were performed on day 1 and recall was tested on day 2.

The experimental method closely resembled the one described in Albayrak et al. (2023), which was based on the earlier study by Milad et al. (2007). To focus on conditioning to the cue rather than the context, we performed fear acquisition and extinction training in two separate contexts (acquisition context and extinction context) represented by pictures of two different office rooms: one with a desk (“office”) and one with a bookshelf (“library”). The CSs were represented by a lamp emitting either blue, red, or yellow light. The lamp was always present in both contexts. The US was administered in the form of an electric shock to the left shin. Two CS+ were presented during fear acquisition training which were both reinforced by an electric shock in 66.67% of the trials. CS− was never paired with the shock. During extinction training, one of the CS+ (extinguished CS+, CS+E) and the CS− were presented. During recall, the extinguished (CS+E), the unextinguished (CS+U), and the CS− were shown. A CS+U was used in addition to a CS+E for direct comparison of recall of extinction (CS+E) and fear (CS+U) during recall (Milad et al., 2007).

The experimental protocol on day 1 consisted of three phases: habituation (2 CS+E only trials, 2 CS+U only trials, 2 CS only trials, presented in acquisition context), fear acquisition training (8 paired CS+E/US trials, 8 paired CS+U/US trials, 4 CS+E only trials, 4 CS+U only trials, 12 CS only trials, presented in acquisition context), and extinction training (12 CS+E only trials, 12 CS only trials, presented in extinction context). The experimental protocol on day 2 consisted of the recall phase (8 CS+E only trials, 8 CS+U only trials, 8 CS only trials, presented in extinction context).

The different trial types in each phase were presented in pseudorandomized order, following two specific restrictions: First, the initial two trials and the final trial during fear acquisition training were CS+ trials reinforced with the US. Second, the number of events of each type was the same in the first and second halves of the experiment. The sequence of events was identical for all participants during habituation and extinction training. During fear acquisition training, the order of CS+E and CS+U events was counterbalanced. During recall, the first event was counterbalanced between CS+E and CS+U trials.

Presentation of the paradigm was managed by a computer running Presentation software (version 21.0, Neurobehavioral Systems). To ensure the synchronization of stimulus presentation with fMRI data acquisition, we sent MRI scan triggers to the control computer.

Images were displayed on a rear projection screen located inside the MRI scanner bore using a standard projector. Participants viewed these images through a mirror attached to the radiofrequency head coil (1chTx/32ChRx coil, Nova Medical).

Electric stimulation was generated using a constant current stimulator (DS7A, Digitimer) and applied to the left shin through a concentric (ring-shaped) bipolar surface electrode with a conductive diameter of 6 mm and a central platinum pin (WASP electrode, Specialty Developments). The electrode position was marked with a permanent marker on day 1 to ensure the same electrode placement on day 2. The 100 ms US consisted of a brief series of four consecutive 500 µs current pulses (maximum output voltage, 400 V) with an interpulse interval of 33 ms. Stimulation intensity was assessed prior to the start of MRI measurements. The stimulation current was incrementally raised while participants were asked to report the perceived sensation until an “unpleasant but not painful” intensity was reached. To mitigate the risk of habituation to the US, which could result in a weakening of the conditioned responses, we increased the final stimulation intensity by 20% (Inoue et al., 2020). The upper limit of the stimulation current was fixed at 40 mA, and this threshold was reached by four participants. The final individual current setting remained the same during the experiment (mean current controls, 7.84 ± 3.10 mA; range, 3.00–14.0 mA; patients, 10.4 ± 14.3 mA; range, 2.20–40.0 mA; independent-samples *t* test, *t*_(39)_ = 1.85; *p* = 0.073).

On both days, immediately prior to the experiment each participant read instructions on the screen stating that they would be shown visual stimuli and that electrical shocks would be applied during the experiment. On day 1, participants were informed that if they detected any pattern between stimuli, that this pattern would not change throughout the experiment. At the beginning of day 2, participants were informed that any pattern perceived during day 1 would stay the same on day 2. Participants confirmed that they had read and understood the instructions.

Each trial included an 8 s presentation of the CS, with the US being presented 7.9 s after the CS onset and co-terminating with the CS in case of reinforced trials. Contexts and CS colors were pseudorandomly counterbalanced across participants. The context image was uninterruptedly displayed throughout each phase. Intertrial intervals (ITIs) were randomized between 12.57 and 15.22 s.

Each experimental phase was performed within a separate session of fMRI data acquisition.

#### Questionnaires

After each phase of the experiment, participants completed a questionnaire (Fig. 1). The questions were displayed on the screen within the MRI scanner and participants provided their responses using a button box held in their right hand.

As outlined in more detail in Batsikadze et al. (2022), participants were asked to rate their (hedonic) valence, (emotional) arousal, fear, and US expectancy on viewing images of the CS+E, CS+U, and CS− on a nine-step Likert scale from “*very pleasant*” to “*very unpleasant*,” “*very calm*” to “*very nervous*,” “*not afraid*” to “*very afraid*,” and “*US not expected*” to “*US surely expected*,” respectively. After fear acquisition training, participants were asked to rate US unpleasantness on a Likert scale from 1 (“not unpleasant”) to 9 (“very unpleasant”) and to estimate mean probability (%) that a US occurred after the CS presentation (CS/US contingency).

At individual time points (prior to, postfear acquisition training, postextinction training, and postrecall), ratings were analyzed using nonparametric ANOVA type statistics with the respective rating as dependent variable, stimulus as within-subjects factor, and group (patients, controls) as between-subjects factor as well as their interactions. No significant differences were expected between the two CS+s in habituation and acquisition. Therefore, prior to and postfear acquisition training, the two CS+s were averaged to reduce degree of freedom.

#### Physiological data acquisition

During the experiment, SCRs and electrocardiogram (ECG) recordings were recorded using MRI-compatible skin conductance and ECG devices, along with the necessary hardware filters (MP160, BIOPAC Systems). The sampling rate was set at 5 kHz. Two skin conductance electrodes were attached to the participants’ left hypothenar, positioned approximately 2 cm apart, while ECG was recorded through three electrodes placed to the participant’s left side of the chest.

#### Skin conductance response evaluation

To eliminate high-frequency noise, skin conductance data was low-pass filtered with a 10 Hz cutoff using a hardware filter (EDA100C-MRI module, BIOPAC Systems). Offline data processing was performed using MATLAB-based (Release 2019a, RRID: SCR_001622, The MathWorks) software EDA-Analysis App (Otto et al., 2023). Data was down sampled to 1 kHz and semiautomated peak detection was performed. SCRs were defined as the maximum trough-to-peak amplitude starting within time intervals from 1 to 8 s after the onset of the CS within the conditioned response window (Pineles et al., 2009) and from 8 to 12 s after CS onset within the unconditioned response window (Prokasy and Ebel, 1967) with a minimum amplitude of 0.01 μs and a minimum rise time of 500 ms (Boucsein et al., 2012). Trials that did not meet the criteria were scored as zero and included in the subsequent data analysis (Pineles et al., 2009).

Raw SCRs were averaged in blocks and normalized through a logarithmic [LN(1 + SCR)] transformation (Venables and Christie, 1980; Boucsein, 2012). Two habituation trials of the same CS were combined to form single blocks. In the subsequent phases, the trials of the same CS were divided into early and late blocks. Specifically, during fear acquisition and extinction training, the averaging included the first and last six trials, while in the recall, the averaging included the first and last four trials. Normality was assessed for the normalized data and the distribution of residuals using the Shapiro–Wilk test. As the normality test revealed a non-normal distribution of SCRs and the residuals (*p *< 0.05), the subsequent data analysis was conducted using nonparametric statistical methods. To this end, we used nonparametric statistical analysis for repeated measures using rank-based *F* tests (ANOVAF option in the PROC MIXED method in SAS, SAS Studio 3.8, SAS Institute) and nparLD R package (http://www.*R*-project.org/), which has been recommended for dealing with skewed distributions, outliers, or small sample sizes. These methods use ANOVA-type statistic with the denominator degrees of freedom set to infinity (Brunner et al., 2002; Noguchi et al., 2012) to enhance the reliability of the ANOVA-type statistic. Using finite denominator degrees of freedom can lead to increased type I errors (Bathke et al., 2009).

To reduce degrees of freedom, CS+E and CS+U trials were averaged in early and late acquisition blocks. Nonparametric ANOVA-type statistics for repeated measures were used separately for each phase with SCRs as dependent variable and stimulus (CS+, CS−) and block (early, late) as within-subjects factors and group (patients, controls) as between-subjects factor as well as their interactions. Throughout the manuscript, in case of significant results of nonparametric ANOVA, post hoc comparisons were performed using least square means tests and were adjusted for multiple comparisons using the Tukey–Kramer method. To quantify the effect sizes, we utilized a metric known as relative treatment effects (RTE). RTE is a measure that falls within the range of 0 to 1. The equation *p_X_ = P(X < Y)* represents the RTE for a specific factor level (*X*) compared with a fixed reference distribution’s mean value (*Y*). If *p_X_* is less than *p_Z_*, it indicates that measurements taken under condition *X* are generally smaller than those under condition Z. Conversely, if *p_X_* = *p_Z_*, it suggests that there is no consistent difference between the data from conditions *X* and *Z*. For example, a *p_X_* value of 0.25 indicates approximately a 25% probability of randomly selecting a subject from the entire dataset who would score lower than a randomly chosen subject from condition *X*.

In addition to the SCR amplitudes, we were interested in the SCR incidences, which are the main output parameter in classical eyeblink conditioning studies (Gerwig et al., 2005; Timmann et al., 2005) and has also been used in Maschke et al. (2002), one of the few studies examining fear conditioning in cerebellar patients. Individual SCR incidences were modeled as a binary (Yes/No) outcome (Daum et al., 1993), and in each block, percentages of SCRs were calculated. Statistical analysis was performed as described above using SCR incidences as dependent variable.

#### Electrocardiogram evaluation

ECG data processing was performed using MATLAB software (Release 2019a, RRID: SCR_001622, The MathWorks). To eliminate high-frequency noise and low-frequency drifts, ECG data were bandpass filtered (0.5–45 Hz). Semiautomated peak detection was performed, and for each trial within the interval from 2 s before to 8 s after CS onset, corresponding beats per minute (bpm) were calculated. Trials were excluded from the analysis in which the ECG peaks could not be detected due to MRI-induced noise or participants’ motion.

Due to poor data quality (excessive number of artifacts caused by MRI electrical noise), identification of ECG peaks was not reliable neither by peak detection algorithms nor manually. Therefore, no ECG data will be presented.

#### MRI acquisition

All MR images were acquired with the participants lying in a headfirst supine position inside a whole-body 7 T MRI scanner (MAGNETOM 7 T, Siemens Healthcare) equipped with a 1-channel transmit 32-channel receive array head coil (Nova Medical). To ensure homogeneity of the radiofrequency excitation field (B1), we positioned three dielectric pads filled with high-permittivity fluid below and on either side of each participant’s upper neck (Teeuwisse et al., 2012). Additional cushions were used as necessary to fix the participants’ head position within the coil.

Functional MRI acquisition was performed to cover the whole brain with an isotropic voxel size of 1.7 mm using a fat-saturated, two-dimensional simultaneous multi-slice echo planar image (SMS-EPI) sequence (Setsompop et al., 2012; Cauley et al., 2014) provided by the Center of Magnetic Resonance Research, University of Minnesota (release R016a). Further imaging parameters were selected as follows: TR/TE, 1999/22.4 ms; flip angle, 70°; parallel acceleration factor (GRAPPA), 2; SMS factor, 3; phase partial Fourier factor, 6/8; acquisition matrix, 130 × 130; number of slices, 93; bandwidth, 1,326 Hz/pixel; and phase encoding direction, posterior to anterior. To correct for distortion artifacts before functional acquisition, we acquired a brief EPI sequence for 5 volumes, with opposite phase encoding direction, that is, anterior to posterior, and otherwise identical parameter settings as the actual fMRI sequence.

After fMRI acquisition on day 1, a sagittal MP2RAGE sequence with fat navigators (Marques et al., 2010; Gallichan and Marques, 2017) was run to acquire anatomical reference images with an isotropic voxel size of 0.60 mm. Further imaging parameters were set as follows: TR/TE, 5,600/2.83 ms; TI1/TI2, 1,000/2,900 ms; flip angles 1/2, 7°/6°; parallel acceleration factor, 3; phase and slice partial Fourier factor, 7/8; acquisition matrix, 370* × *370; number of slices, 288; and TA, 12:06 min.

#### Image processing

All image and fMRI analysis were performed on a 64 bit Linux machine, predominantly using MATLAB (R2019a) and SPM12 (Wellcome Department of Cognitive Neurology), as well as and the Advanced Normalization Tools (ANTs, version 2.3.5; Tustison et al., 2014).

Based on fat navigators, motion correction for MP2RAGE volumes was performed using offline reconstruction with the Retro-MoCo toolbox for MATLAB provided by David Gallichan (version 0.9.0dev; https://github.com/dgallichan/retroMoCoBox.git). Motion-corrected T1-weighted image volumes were denoised, and dielectric bags were removed from the volumes using the presurfer toolbox for SPM12 (DOI: 10.5281/zenodo.4626841) and the ANTs-based ants_deface_depad tool (DOI: 10.5281/zenodo.4626833) both written by Sriranga Kashyap. Normalization parameters into MNI space were generated using the segmentation function of the Computational Anatomy Toolbox for SPM12 (CAT12, release 1450; Gaser et al., 2022).

Using ANTs, each functional run was motion corrected, distortion corrected using the opposed phase EPI sequence described above, and finally registered to the denoised and de-padded anatomic volume. Subsequently using SPM, normalization into MNI space derived from CAT12 segmentation was applied on fMRI data. SPM-like motion regressor tables (3 translations, 3 rotations) were derived from ANTS affine transformation matrices output.

#### Voxel-based morphometry (VBM)

For the VBM analysis, the CAT12 toolbox was used in SPM12 as outlined in the image processing section (CAT12, release 1450; Gaser et al., 2022). The default settings for CAT12 were used, with the inclusion of gray matter (GM) modulation to compensate for the effects of spatial normalization. The CAT12 toolbox first corrects for bias field inhomogeneities on the denoised MP2RAGE images and then performs affine and nonlinear registration to MNI-space. The output of CAT12 includes GM, white matter (WM), and cerebrospinal fluid (CSF) probability maps in MNI space, as well as GM, WM, and CSF segmentations in native space. The GM probability maps in MNI space were smoothed with an 8-mm-wide Gaussian kernel. The total intercranial volume (TIV) was determined as the sum of the volume of GM, WM, and CSF segmentations in native space. The smoothed maps were used for second-level analysis in SPM12, with cerebellar patient and control groups included in the design matrix (two-sample *t* test), along with age and TIV as covariates of no interest. The resulting morphometry maps were masked using the SUIT atlas volume (Cerebellum-SUIT.nii) with the inner cerebellar white matter manually filled in. To display results, we plotted morphometry maps on cerebellar flatmaps (Diedrichsen and Zotow, 2015) using TFCE and family-wise error (FWE) correction (*p* < 0.05).

#### fMRI analysis

The first-level analysis was performed on the normalized but unsmoothed fMRI data, and a cerebellar mask, specifically the SUIT atlas volume (Cerebellum-SUIT.nii) with the inner cerebellar white matter manually filled in, was applied. The entire experiment was modeled in a first-level analysis as an event-related design, wherein all event durations were set to 0 s. Movement parameters resulting from volume realignment were added as regressors of no interest. The onsets of presentations for the CS+E, CS+U, CS−, and US (including the corresponding timing for unpaired trials, i.e., US omission after CS presentation, further referred to as no-US) were modeled as individual events. Individual events were blocked as shown in Figure 1. If the number of events per phase allowed, that is, *n* > 5, the experimental phases were split into an early and a late block. The first-level main effect contrasts against baseline and relevant differential first-level contrasts were computed, smoothed with a smoothing kernel of 5.1 mm (3 times acquisition voxel size), and tested in second-level *t* tests. In accordance with a previous study using a comparable paradigm in young and healthy controls (Batsikadze et al., 2022), we decided to report the data of CS+ and CS− activations against the baseline instead of using differential contrasts.

Threshold-free cluster enhancement (TFCE) was applied using the TFCE toolbox for SPM12 (R174; http://dbm.neuro.uni-jena.de/tfce/). To display results, we plotted cerebellar (SUIT space) activation maps on cerebellar flatmaps (Diedrichsen and Zotow, 2015) using TFCE and FWE correction (*p *< 0.05). Activation maps were projected onto the SUIT atlas volume (Cerebellum-SUIT.nii) (Diedrichsen, 2006) to acquire anatomical region labels.

### Animal study

**Figure 2.**
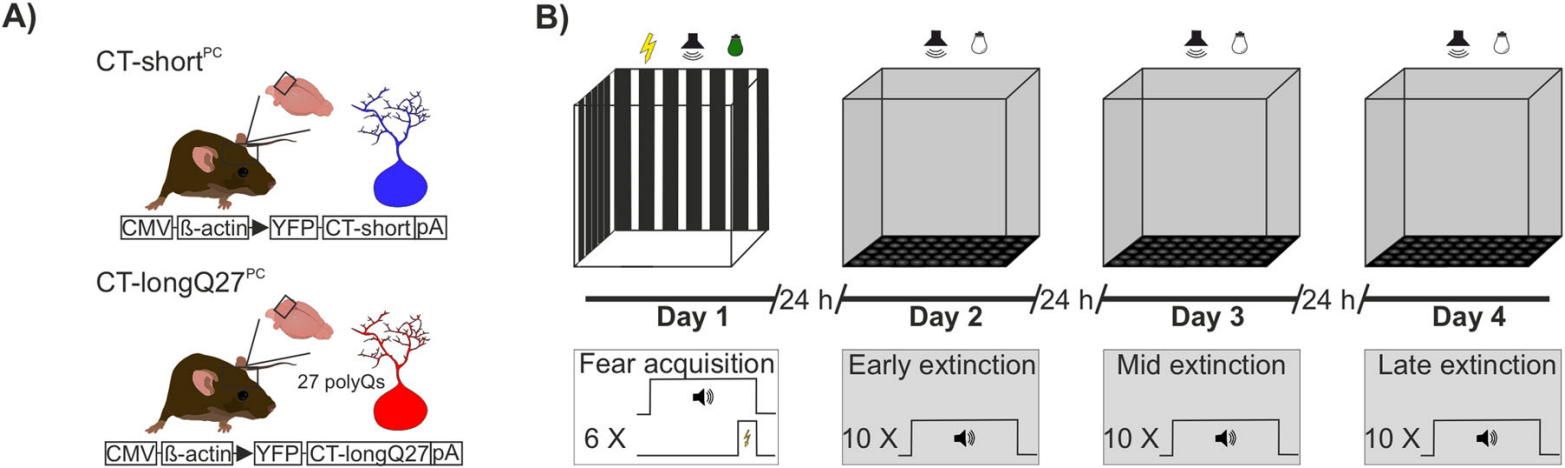
Experimental paradigm for animal study. ***A***, The mouse models expressed the human C terminus with 27 polyQ repeats in Purkinje cells (CT-longQ27^PC^), displayed in red and their corresponding CT-short^PC^ controls in blue which lack polyQ repeats. ***B***, Schematic representation of the auditory fear conditioning protocol consisting of fear acquisition training, during which six tone and foot shock pairings were given, followed by three extinction training days, during which 10 tones each were presented.

For comparison, fear conditioning was studied in a mouse model of cerebellar cortical degeneration (i.e., SCA6). We analyzed fear learning using an auditory pavlovian fear conditioning paradigm in SCA6 degenerated CT-longQ27^PC^ and control CT-short^PC^ mice at three different ages (pre-onset, early stage, and late stage; Fig. 2*A*).

#### Animals

Mice were housed in groups of 2–3 animals per cage with food and water ad libitum. Two weeks before behavioral testing, mice were isolated in a separate room that had a constant temperature of 22°C with a 12 h light/dark cycle. Experiments were performed during the light phase. Fear conditioning was performed in mice with SCA6-like symptoms which were previously described by Mark et al. (2015). By crossing floxed CT-longQ27 or CT-short mice with transgenic Purkinje cell-specific cre mice [Jackson Laboratories, stock number 004146 B6.129-Tg (Pcp2-cre)2Mpin/J; Barski et al., 2000], Purkinje cell-specific expression of CT-longQ27^PC^ and CT-short^PC^ mice, respectively, were created. Transgene expression was detected by polymerase chain reaction analysis for CT-short and CT-longQ27 forward, 5′ CGACCACTACCAGCAGAACA 3′, reverse, 5′ CCACGGACTGAGAGTTAGGC 3′ and Tg-cre forward, 5′ ATTCTCCCACCACCGTCAGTACG 3′, reverse, 5′ AAAATTTGCCTGCATTACCG 3′. Male (M) and female (F) mice were used for the experiments and divided into three age groups: pre-onset, 3–5 months old for CT-short^PC^ (6 M, 4F) and CT-longQ27^PC^ (6 M, 4F); early stage, 7–9 months old for CT-short^PC^ (7 M, 3F) and CT-longQ27^PC^ (4 M, 6F); and late stage, >12 months old for CT-short^PC^ (8 M, 7F) and CT-longQ27^PC^ (4 M, 11F).

Data acquisition was conducted in at least two separate fear conditioning experiments. Experiments were authorized by the local ethics committee (Bezirksamt Arnsberg) and the animal care committee of LANUV (Landesamt für Umweltschutz, Naturschutz und Verbraucherschutz Nordrhein-Westfalen, Germany). The studies were conducted following the European Communities Council Directive of 2010 (2010/63/ EU) for the care of laboratory animals and supervised by the animal welfare committee of the Ruhr-University Bochum. All efforts were made to minimize the number of mice used for this study.

#### Fear conditioning paradigm

Auditory fear conditioning was performed in an acrylic glass chamber (23 cm × 25 cm × 24 cm) which was shielded from external noise by a noise-reducing cabinet (background noise, 35 ± 5 dB). To ensure conditioning to the auditory cue and not to the context, we performed fear acquisition and extinction training in two contexts which differed in light, texture, odor, and visual surrounding (Fig. 2*B*). The acquisition context incorporated a darkened experimental room and a fear conditioning chamber with black and white striped walls. The bottom of the chamber was equipped with a foot shock grid. The chamber was lit with infrared and green (65 lux) light and scented with 0.05% Helipur (B. Braun). The extinction context consisted of a brightly lit experimental room, and the chamber had gray-colored walls and texturized flooring with infrared illumination. The chamber was slightly scented with 70% EtOH. The cabinet was equipped with a speaker (FR 58 VISATON) to deliver the sound and a video camera (Mako U-130B Allied Vision Technologies) to enable post hoc analysis of fear behavior.

The fear conditioning setup was controlled by a custom-written MATLAB (The MathWorks) script. Prior to behavioral testing, mice were habituated to handling by the scientist. To reduce stress, cage changes were always performed by the same scientist, and the last cage change was performed 3 d before behavioral testing. Fear acquisition training took place on the first day in the acquisition context. The mice were placed into the fear conditioning chamber, and a 2 min baseline period was followed by 6 tone/shock pairings (CS 30 s, 7.5 kHz, 60 dB/US 2 s 0.45 mA co-terminating with the CS). The time between CS presentations (ITI) varied between 60 and 180 s. The fear conditioning chamber was cleaned with soap and water after each animal. After a 24 h consolidation period, the mice were brought to the extinction context for fear extinction training. After placing the animal into the chamber, a 2 min baseline period was followed by 10 tones (CS 30 s, 7.5 kHz, 60 dB). ITIs varied between 30 and 180 s. Extinction training was repeated on 3 consecutive days (early, mid, and late extinction) to ensure complete fear extinction.

#### Freezing response evaluation

Freezing behavior was later analyzed by EthoVision XT 11.5 (Noldus Information Technology). Fear learning-related freezing behavior was analyzed during the 30 s time interval of CS presentation. Analysis of extinction training sessions contained the 2 min baseline (B) period and the 30 s time intervals of CS presentation. Retrieval was defined as freezing responses to the first two CS presentation of early extinction. Retrieval is therefore a measure of fear learning during acquisition training. Note that retrieval and recall are often used synonymously. In our human experiments, however, recall was defined as fear responses in unreinforced CS+ trials presented on the second day, which followed acquisition and extinction training on the first day. In recall, occurrence of fear responses is therefore a measure of spontaneous recovery following extinction training. To further investigate the behavior during retrieval, we additionally plotted the percentage of animals of each group which displayed freezing levels below 20%.

Freezing was defined as the absence of movement with the exception of respiratory movement. Based on these criteria, the analysis settings were chosen in EthoVision XT. The behavior of the animal was assessed as freezing if the pixel change from one frame to another was below 0.07/0.02% (acquisition/early, mid, and late extinction) for at least 2 s. The sample rate of the recorded videos was 24.99 frames/s, and the activity threshold was set to 9 with a noise filter of 2 and compression artifacts filter. The analysis was manually verified.

Shapiro–Wilk test was used to test the freezing data and the distribution of residuals for normality. Since Shapiro–Wilk test revealed violation of normality, a nonparametric statistical analysis was used. Nonparametric two-way ANOVA-type statistics (ANOVAF option in the PROC MIXED method in SAS, SAS Studio 3.8, SAS Institute, and nparLD R package; http://www.*R*-project.org/) for repeated measures were used separately for acquisition, early, mid, and late extinction with freezing behavior as dependent variable, genotype (CT-longQ27^PC^ and CT-short^PC^) as between-subjects factor, and trial (acquisition CS trial 1–6/extinction CS trial 1–10) as within-subjects factor as well as their interactions. Due to technical problems, one early stage CT-short^PC^ animal was not video recorded during the mid extinction session and therefore could not be included in the analysis of the mid extinction session. However, since it still successfully completed the session, it was not excluded from the remaining fear conditioning paradigm. Fear-related changes between baseline and retrieval freezing were conducted by nonparametric two-way ANOVA-type statistics for repeated measures separately for the respective age groups (Extended Data Table 12-2) with freezing behavior as dependent variable, genotype (CT-longQ27^PC^ and CT-short^PC^) as between-subjects factor, and trial (baseline vs retrieval) as within-subjects factor as well as their interactions and followed up by post hoc analysis (Extended Data Table 12-3). Statistical analyses were conducted with SAS. Statistical significance was set at *p *< 0.05.

## Results

### Human study

#### Depression-Anxiety-Stress-Scale-21

**Figure 3.**
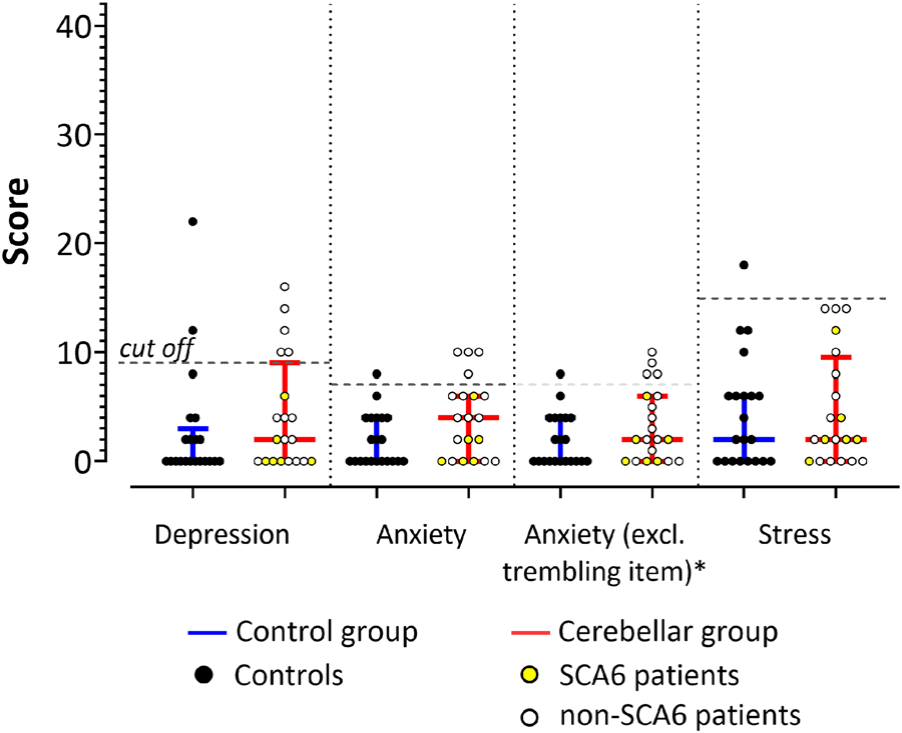
Results of DASS-21 questionnaire. Median scores and interquartile range (IQR) in the patient and control groups. The DASS-21 questionnaire scores did not reveal any significant group differences (Mann–Whitney *U* test). Horizontal lines denote median values; whiskers range from the first to the third quartile. Normal range: depression score, 0–9; anxiety score, 0–7; stress score, 0–14; maximum possible score, 42 (Lovibond et al., 1995). Circles represent individual scores: yellow circles represent SCA6 patients; white circles, non-SCA6 patients; black circles—controls. For color-coding of individual patients based on SARA score see Extended Data Figure 3-1. *The questionnaire results were reanalyzed excluding the trembling-related item.

Five (25%) patients and two (9.5%) control participants showed mildly to moderately elevated depression scores. Four (20%) patients and one control (4.8%) showed mildly to moderately elevated anxiety scores, and one control (4.8%) showed mildly to moderately elevated stress scores. Stress (*U* = 197.5; *p* = 0.757; Mann–Whitney *U* test) and depression scores (*U* = 163.5; *p* = 0.23) were not significantly different between groups. Anxiety scores were higher in patients, but the difference did not reach statistical significance (*U* = 138.5; *p* = 0.064). Of note, one question in the anxiety subscale of the DASS21 questionnaire asks whether the participants experienced trembling (e.g., in the hands), and patients may report cerebellar tremor. We therefore decided to reanalyze the anxiety subscale by excluding the trembling-related question (in patients and controls). Difference between groups was less pronounced (*U* = 154.5; *p* = 0.153), and the number of controls and patients performing in the abnormal range was unchanged (Fig. 3).

10.1523/ENEURO.0365-23.2023.f3-1 Figure 3-1 Results of Depression-Anxiety-Stress-Scale-21 (DASS-21) questionnaire. Median scores and interquartile range (IQR) in the patient and control groups. The DASS-21 questionnaire scores did not reveal any significant group differences (Mann-Whitney-U test). Horizontal lines denote median values, whiskers range from the first to the third quartile. Normal range: depression score: 0-9, anxiety score: 0-7, stress score: 0-14; maximum possible score: 42 (Lovibond et al., 1995). Colored circles represent individual responses of patients based on ataxia severity (Lai et al., 2019; Yang et al., 2020): green circles represent patients with mild ataxia (SARA score ≤ 10, *n* = 5), yellow circles = patients with moderate ataxia (SARA score > 10 and < 20, *n* = 12), orange circles = patients with severe ataxia (SARA score ≥ 20, *n* = 3), black circles = controls. Download Figure 3-1, TIF file.

#### Behavioral fear conditioning data

##### Questionnaires

*Valence, arousal, fear.* Nonparametric ANOVA-type statistic revealed a significant main effect of stimulus (CS+E vs CS+U vs CS−), time (prior acquisition training vs postacquisition training vs postextinction training vs recall), and a stimulus × time interaction for all three ratings (all *p* values <0.001). The group main effects and all interactions were not significant (see Extended Data Table 4-1 for summary of nonparametric ANOVA-type statistical analysis). RTE estimates are shown in Extended Data Figure 4-1.

**Figure 4.**
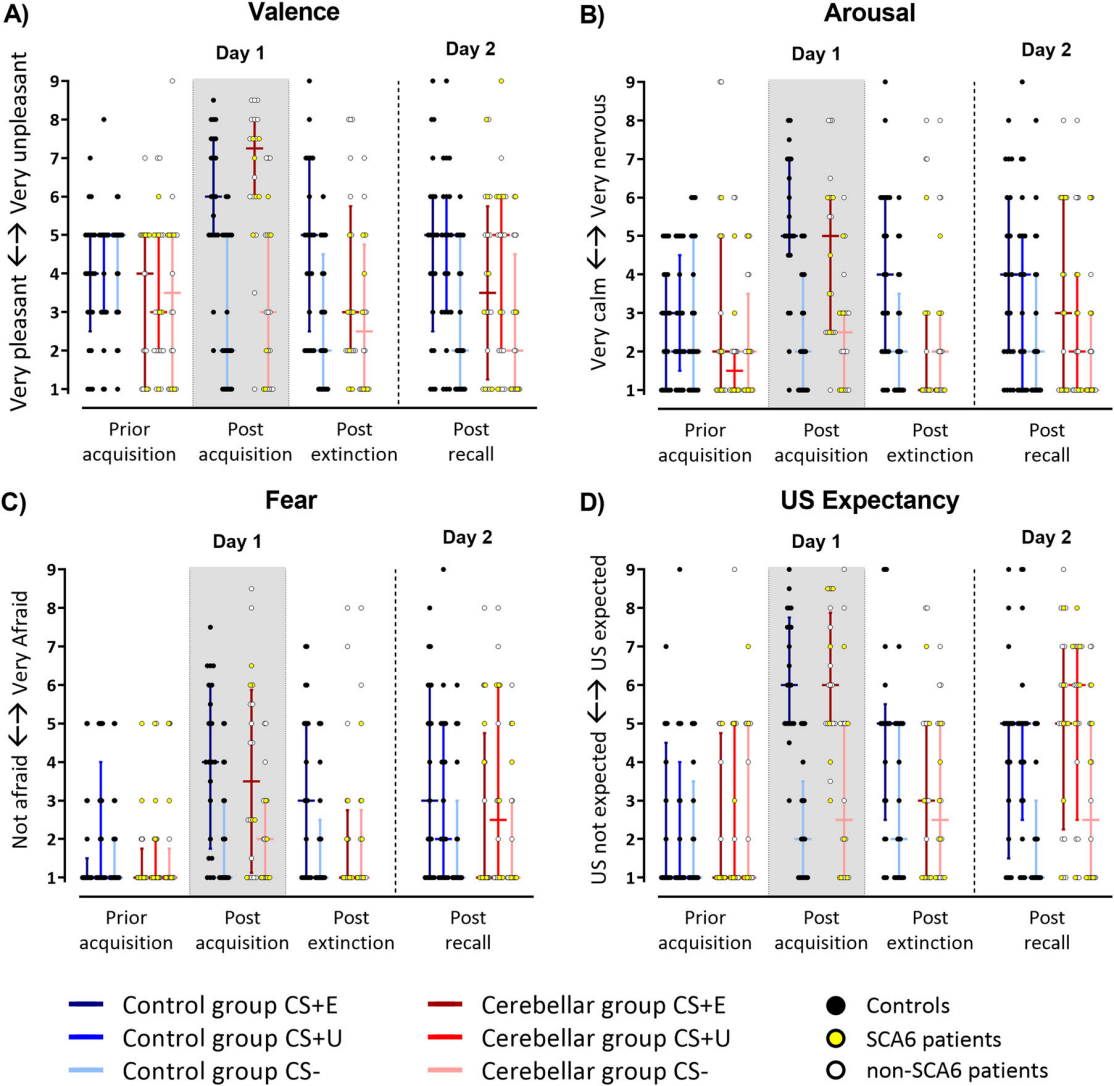
Results of questionnaires in cerebellar patients and controls prior acquisition, postacquisition, postextinction, and postrecall. Median ratings regarding (***A***) valence, (***B***) arousal, (***C***) fear, and (***D***) US expectancy on a Likert scale of 1 (“*very pleasant*”/“*very calm*”/“*not afraid*,” “*US not expected*,” respectively) to 9 “*very unpleasant*”/“*very nervous*”/“*very afraid*,” “*US expected*,” respectively). Horizontal lines denote median values. Whiskers range from the first to the third quartile. Blue colors, controls; red colors, cerebellar patients. Dark colors, CS+E and CS+U; light colors, CS–. Circles represent individual responses: yellow circles represent SCA6 patients; white circles, non-SCA6 patients; black circles, controls. For color-coding of individual patients based on SARA score, see Extended Data Figure 4-2. Gray background, fear acquisition training. Postacquisition training responses to CS+E and CS+U were averaged (CS+_avg_). Both controls and patients showed differential responses toward the CS+ and CS− postacquisition and postrecall. This difference was also present postextinction in controls, but not in patients. RTE estimates are shown in Extended Data Figure 4-1; statistical findings are summarized in Extended Data Table 4-1.

Post hoc nonparametric ANOVA-type statistics on individual time points revealed that both the postacquisition (averaged) and postrecall valence of the CS+ were significantly less pleasant, and arousal and fear were rated significantly higher compared with the CS− (Fig. 4*A–C*, all *p* values ≤0.02). Postextinction valence, arousal, and fear ratings showed significant stimulus × group interactions (valence, *p* = 0.020; arousal, *p* = 0.013; fear, *p* = 0.027). Controls reported significantly more negative valence, higher arousal, and fear toward CS+E compared with the CS− (least squares means tests; all *p* values ≤0.033), whereas cerebellar patients did not rate them differently (least squares means tests; all *p* values ≥0.985).

10.1523/ENEURO.0365-23.2023.f4-1 Figure 4-1 Relative treatment effect (RTE) estimates for **A)** valence, **B)** arousal, **C)** fear and **D)** US expectancy. Horizontal lines denote median RTEs and whiskers denote 95% confidence intervals. Blue colors = controls, red colors = cerebellar patients. Dark colors: CS+E and CS+U, light colors: CS–. Gray background = fear acquisition training. Post acquisition training responses to CS+E and CS+U were averaged (CS+_avg_). Download Figure 4-1, TIF file.

10.1523/ENEURO.0365-23.2023.f4-2 Figure 4-2 Results of questionnaires in cerebellar patients and controls prior acquisition, post acquisition, post extinction and post recall. Median ratings regarding **A)** valence, **B)** arousal, **C)** fear and **D)** US expectancy on a Likert-scale of 1 (*“very pleasant”/“very calm”/“not afraid”, “US not expected”*, respectively) to 9 *“very unpleasant”/“very nervous”/“very afraid”, “US expected”*, respectively). Horizontal lines denote median values. Whiskers range from the first to the third quartile. Blue colors = controls, red colors = cerebellar patients. Dark colors: CS+E and CS+U, light colors: CS–. Colored circles represent individual responses of patients based on ataxia severity (Lai et al., 2019; Yang et al., 2020): green circles represent patients with mild ataxia (SARA score ≤ 10, *n* = 5), yellow circles = patients with moderate ataxia (SARA score > 10 and < 20, *n* = 12), orange circles = patients with severe ataxia (SARA score ≥ 20, *n* = 3), black circles = controls. Download Figure 4-2, TIF file.

10.1523/ENEURO.0365-23.2023.t4-1 Table 4-1 Results of the non-parametric ANOVA-type statistics for repeated measures for valence, arousal, fear and US expectancy ratings comparing cerebellar patient and control groups. Download Table 4-1, DOC file.

*US unpleasantness, US expectancy, CS–US contingency.* Cerebellar and control participants both rated the US as unpleasant, and unpleasantness was not different between groups [postacquisition training: patients, median 7 (IQR 6–8); controls, median 7 (IQR 5.75–7.25); on a Likert scale from 1 (not unpleasant) to 9 (very unpleasant); Mann–Whitney *U* test; *U* = 188.5; *p* = 0.582].

Prior to fear acquisition training, US expectancy ratings after CS+E, CS+U, and CS− presentations were not different. Postacquisition (averaged) and postrecall US expectancy after the CS+ were rated significantly higher compared with the CS− with no significant differences between groups (least squares means tests; all *p* values <0.001; Fig. 4*D*; Extended Data Table 4-1); that is, both groups learned the CS/US contingencies. Nonparametric ANOVA-type statistic revealed a significant main effect of stimulus (CS+E vs CS+U vs CS−), time (prior fear acquisition training vs postfear acquisition training vs postextinction training vs postrecall), and a stimulus × time interaction (all *p* values <0.001; Extended Data Table 4-1). Postextinction training, post hoc nonparametric ANOVA-type statistics revealed a significant stimulus × group interaction (*p* = 0.024) with controls rating US expectancy after the CS+E significantly higher than that after CS− (least squares means test; *p* = 0.02), while patients did not (least squares means test; *p* = 0.862).

Postacquisition, controls reported an estimated mean probability that a US occurring following CS presentation as 55.7 ± 26% after CS+E, 52.4 ± 28.8% after CS+U, and 5.7 ± 12.9% [0% probability by 16 out of 21 (76%) controls] after CS− presentation. Cerebellar patients reported mean probability that a US occurred after presentation of CS was estimated as 49.5 ± 24.2% after CS+E, 47.5 ± 30.24% after CS+U, and 16.5 ± 26.3% [0% probability by 12 out of 20 (60%) patients] after CS− presentation. No significant group differences occurred between reported mean probabilities (Mann–Whitney *U* tests; all *p* values ≥0.28).

Postacquisition training, one control (4.76%) and three cerebellar patients (15%) reported that they did not recognize a pattern between CS+ and US presentations. The rest of participants reported that they identified a pattern regarding the contingency between CS+ and US after 5.15 ± 3.75 min. The reported time was significantly longer in cerebellar patients compared with that in controls (controls, 3.85 ± 2.44; patients, 6.67 ± 4.47; independent-samples *t* test, *t*_(35)_ = 1.85; *p = *0.01).

##### Skin conductance response amplitudes

**Figure 5.**
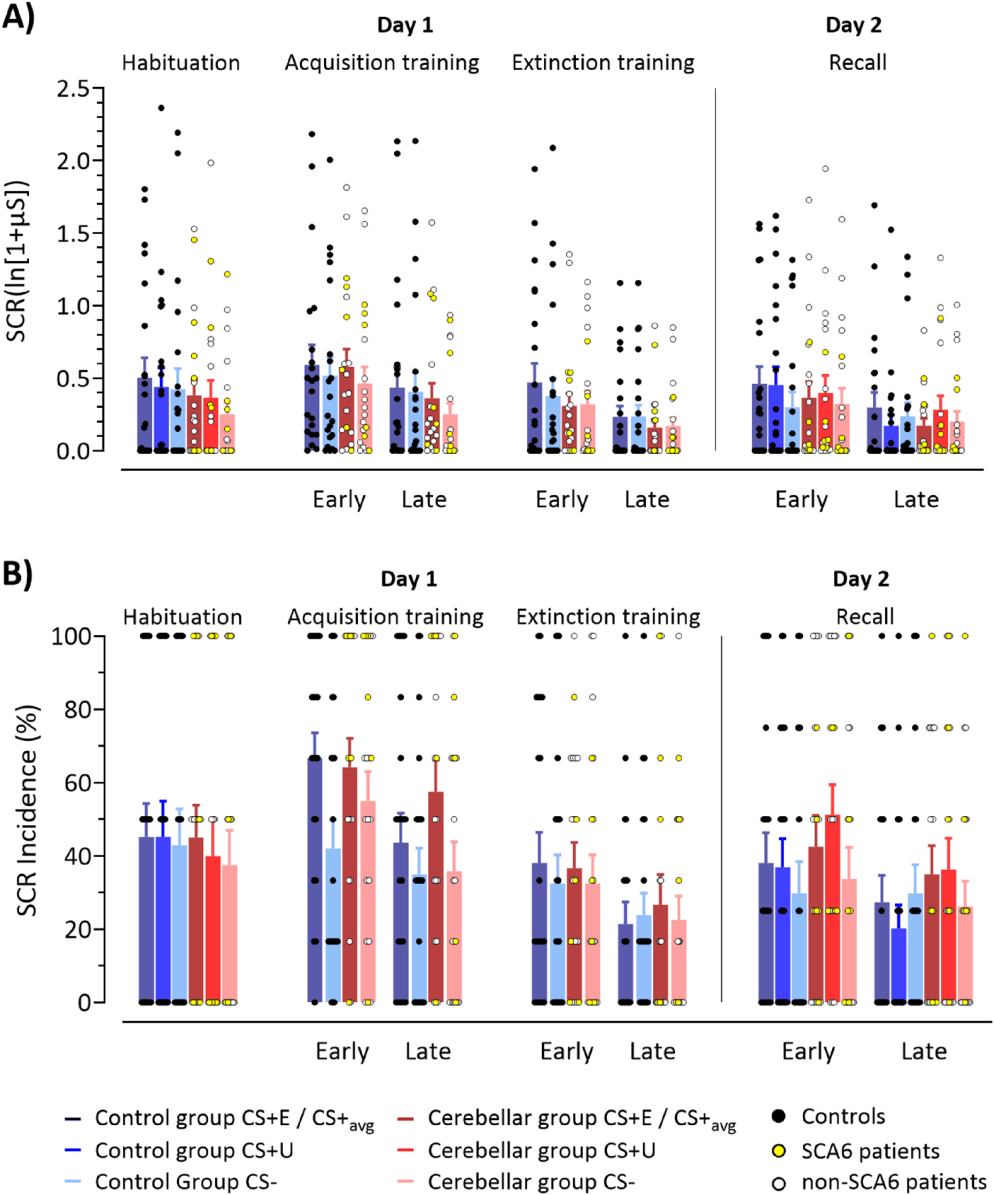
Results of SCRs in cerebellar patients and controls prior acquisition (habituation), during acquisition, extinction, and recall. ***A***, SCR amplitudes and (***B***) SCR incidences. Colored bars represent group mean values for habituation, early and late blocks of fear acquisition training, extinction training, and recall. Postacquisition training responses to CS+E and CS+U were averaged (CS+_avg_). Error bars indicate SEM. Blue colors, controls; red colors, cerebellar patients. Dark colors, CS+E/CS+_avg_ and CS+U; light colors, CS−. Circles represent individual responses: yellow circles represent SCA6 patients; white circles, non-SCA6 patients; black circles—controls. For color-coding of individual patients based on SARA score, see Extended Data Figure 5-3. SCR amplitudes were higher toward the CS+ compared with those toward the CS− in acquisition training and early recall in both patients and controls. In controls, SCR incidences were significantly higher toward the CS+ compared with those toward the CS, already during the early block, but in patients only during the late acquisition block. In recall, SCR incidences were higher for both CS+ trials compared with those for CS− in both groups. Notably, in patients, SCR incidences for CS+U remained significantly higher compared with those for the CS− throughout the entire phase. RTE estimates are shown in Extended Data Figure 5-1; SCR amplitudes related to the aversive US are shown in Extended Data Figures 5-2 and 5-4. Statistical findings are summarized in Extended Data Tables 5-1 (SCR amplitudes) and 5-2 (SCR incidences).

*Habituation phase (day 1)*: Mean SCR amplitudes toward the CS+E compared with those toward the CS+U and CS− did not differ between groups (Fig. 5*A*, Extended Data Table 5-1). Nonparametric ANOVA-type statistics revealed no significant main effects of stimulus (*p* = 0.114), group (*p *=* *0.640), or stimulus × group (*p* = 0.896) interactions.

10.1523/ENEURO.0365-23.2023.f5-1 Figure 5-1 Relative treatment effect (RTE) estimates for **A)** skin conductance responses and **B)** skin conductance response incidences. Horizontal lines denote median RTEs and whiskers denote 95% confidence intervals. Blue colors = controls, red colors = cerebellar patients. Dark colors: CS+E and CS+U, light colors: CS–. Gray background = fear acquisition training. Post acquisition training responses to CS+E and CS+U were averaged (CS+_avg_). Download Figure 5-1, TIF file.

10.1523/ENEURO.0365-23.2023.f5-2 Figure 5-2 Skin conductance response amplitudes (SCRs) related to US presentation and US omission (no-US) after CSs during fear acquisition training; Colored bars represent group mean (log-transformed) values for fear acquisition training. Error bars indicate S.E.M. Blue colors = controls, red colors = cerebellar patients. Full bars = trials followed by the US, striped bars = trials not followed by the US. Dark colors: CS+_avg_ light colors: CS-. Circles represent individual responses: yellow circles represent SCA6 patients, white circles – non-SCA6 patients, black circles - controls. Download Figure 5-2, TIF file.

10.1523/ENEURO.0365-23.2023.f5-3 Figure 5-3 Results of skin conductance responses in cerebellar patients and controls prior acquisition (habituation), during acquisition, extinction and recall. **A)** Skin conductance response (SCR) amplitudes and **B)** skin conductance response incidences. Colored bars represent group mean values for habituation, early and late blocks of fear acquisition training, extinction training and recall. Post acquisition training responses to CS+E and CS+U were averaged (CS+_avg_). Error bars indicate S.E.M. Blue colors = controls, red colors = cerebellar patients. Dark colors: CS+E/CS+_avg_ and CS+U light colors: CS-. Colored circles represent individual responses of patients based on ataxia severity (Lai et al., 2019; Yang et al., 2020): green circles represent patients with mild ataxia (SARA score ≤ 10, *n* = 5), yellow circles = patients with moderate ataxia (SARA score > 10 and < 20, *n* = 12), orange circles = patients with severe ataxia (SARA score ≥ 20, *n* = 3), black circles = controls. Download Figure 5-3, TIF file.

10.1523/ENEURO.0365-23.2023.f5-4 Figure 5-4 Skin conductance response amplitudes (SCRs) related to US presentation and US omission (no-US) after CSs during fear acquisition training; Colored bars represent group mean (log-transformed) values for fear acquisition training. Error bars indicate S.E.M. Blue colors = controls, red colors = cerebellar patients. Full bars = trials followed by the US, striped bars = trials not followed by the US. Dark colors: CS+_avg_ light colors: CS-. Colored circles represent individual responses of patients based on ataxia severity (Lai et al., 2019; Yang et al., 2020): green circles represent patients with mild ataxia (SARA score ≤ 10, *n* = 5), yellow circles = patients with moderate ataxia (SARA score > 10 and < 20, *n* = 12), orange circles = patients with severe ataxia (SARA score ≥ 20, *n* = 3), black circles = controls. Download Figure 5-4, TIF file.

10.1523/ENEURO.0365-23.2023.t5-1 Table 5-1 Results of the non-parametric ANOVA-type statistics for repeated measures for skin conductance response (SCR) amplitudes comparing cerebellar patient and control groups. Download Table 5-1, DOC file.

10.1523/ENEURO.0365-23.2023.t5-2 Table 5-2 Results of the non-parametric ANOVA-type statistics for repeated measures for skin conductance response (SCR) incidences between patient and control groups. Download Table 5-2, DOC file.

*Fear acquisition training (day 1)*: Both groups showed significantly higher mean SCR amplitudes to the averaged CS+ (CS+_avg_) compared with the CS− (Fig. 5*A*). Nonparametric ANOVA-type statistics yielded significant main effects of stimulus (CS+_avg_ vs CS−; *F*_(1)_ = 14.13; *p *<* *0.001) and block (early vs late phase; *F*_(1)_ = 23.77; *p *<* *0.001), but not group (*p* = 0.850) or any of the interactions (all *p* values ≥0.187). RTE estimates are shown in Extended Data Figure 5-1A.

*Extinction training (day 1):* In early extinction, mean SCR amplitudes to the CS+E were numerically higher compared with those to CS− in controls, but not in cerebellar patients (Fig. 5*A*). The size of SCRs was smaller in late compared with that in early extinction (Fig. 5*A*). Nonparametric ANOVA-type statistics revealed a significant main effect of block (early vs late phase; *F*_(1)_ = 11.03; *p* < 0.001). The stimulus (*p* = 0.308) and group (*p *=* *0.857) main effects and all interactions were not significant: stimulus* × *block (*p *=* *0.158), group* × *stimulus (*p *=* *0.904), group × block (*p *=* *0.675), or group* × *stimulus* × *block (*p *=* *0.906).

*Recall (day 2):* In early recall, SCR amplitudes were higher in CS+E and CS+U blocks compared with those in CS− blocks in both groups. In late recall, the difference in SCR amplitudes between CS+s and CS− declined in both groups (Fig. 5*A*). Nonparametric ANOVA-type statistics revealed significant main effects of stimulus (CS+E vs CS+U vs CS−; *F*_(1.92)_ = 4.68; *p* = 0.010), block (early vs late phase; *F*_(1)_ = 11.51; *p* < 0.001), and a significant block* × *stimulus interaction (*F*_(1.87) _= 3.74; *p* = 0.027). The group main effect (*p *=* *0.770) and all other interaction effects were not significant [group* × *stimulus (*p *=* *0.108), group* × *block (*p *=* *0.892), or group* × *stimulus* × *block (*p *=* *0.427)]. In early recall, SCRs for CS+E and CS+U were significantly different from CS− in both groups (all *p* values ≤ 0.002; least squares means test). No difference between CS+E and CS+U was revealed (*p* = 0.992; least squares means test). SCRs in CS+E and CS+U trials were higher in early vs late recall (all *p* values ≤ 0.023; least squares means test), but not toward the CS− (*p* = 0.999; least squares means test). Thus, both groups showed spontaneous recovery of SCRs toward the CS+E, with responses not being different to the CS+U.

Statistical findings are summarized in Extended Data Table 5-1.

##### SCR incidences

*Habituation (day 1)*: Mean SCR incidences did not differ between CS+s compared with CS− in both groups (Fig. 5*B*, Extended Data Table 5-2). Nonparametric ANOVA-type statistics revealed no significant main effects of stimulus (*p* = 0.505), group (*p *=* *0.779), or stimulus* × *group (*p* = 0.818) interactions.

*Fear acquisition training (day 1)*: Both groups showed significantly higher SCR incidences toward the averaged CS+ (CS+_avg_) compared with the CS− in both early and late blocks. Nonparametric ANOVA-type statistics yielded significant main effects of stimulus (CS+_avg_ vs CS−; *F*_(1)_ = 46.99; *p *<* *0.001), block (early vs late phase; *F*_(1)_ = 24.66; *p *<* *0.001), and a significant stimulus × block × group interaction (*F*_(1)_ = 8.28; *p *=* *0.004). The main effect of group (*p* = 0.534) and other interactions (all *p* values ≥0.697) were not significant. Post hoc exploratory analysis of the stimulus × block × group differences revealed significantly higher SCR incidences in the early CS+ compared with early CS− and late CS+ block in controls (all *p* values ≤0.004). In patients, SCR incidences were significantly higher in late CS+ blocks compared with those in late CS− blocks (*p *=* *0.001), as well as early CS− blocks compared with the late CS− blocks (*p *=* *0.02). RTE estimates are shown in Extended Data Figure 5-1*B*.

*Extinction training (day 1):* SCR incidences did not differ toward the CS+E compared with the CS− in both groups (Fig. 5*B*). Nonparametric ANOVA-type statistics revealed significant main effects of block (early vs late phase; *F*_(1) _= 12.33; *p* < 0.001). The group (*p *=* *0.998) and stimulus (*p* = 0.286) main effects and all interactions were not significant: stimulus* × *block (*p *=* *0.102), group* × *stimulus (*p *=* *0.396), group* × *block (*p *=* *0.717), or group × stimulus* × *block (*p *=* *0.514).

*Recall (day 2):* In recall, SCR incidences were higher in CS+E and CS+U blocks compared with those in CS− blocks in both groups (all *p* values ≤ 0.047; least squares means test; Fig. 5*B*). Nonparametric ANOVA-type statistics revealed significant main effects of stimulus (CS+E vs CS+U vs CS−; *F*_(1.9)_ = 4.02; *p* = 0.019), block (early vs late phase; *F*_(1)_ = 7.29; *p* = 0.007), and a significant group* × *stimulus interaction (*F*_(1.9)_ = 3.33; *p* = 0.038). The group main effect (*p *=* *0.418) and all other interaction effects were not significant [stimulus* × *block (*p* = 0.059), group* × *block (*p *=* *0.848), or group* × *stimulus* × *block (*p *=* *0.585)].

Post hoc exploratory analysis of the stimulus* × *group differences revealed higher SCR incidences in CS+U blocks compared with CS− in patients throughout the recall phase (*p = *0.001).

*SCRs related to the aversive US*: Mean SCR amplitudes in the unconditioned response window were higher in the paired CS+_avg_ trials (direct response to the US) compared with the unpaired CS+_avg_ (US omission) and CS− trials (no-US) with no difference between both groups. Nonparametric ANOVA-type statistics revealed a significant main effect of stimulus (paired CS+_avg_ vs unpaired CS+_avg_ vs CS−; *F*_(1.87)_ = 106.04; *p *<* *0.001). The main effects of group (*p *=* *0.886) and group* × *stimulus (*p *=* *0.475) were not significant. SCRs to the US in unpaired CS+_avg_ and CS− trials were not significantly different (*p* = 0.944; least squares means test; Extended Data Figs. 5-2 and 5-4 for color-coding of individual patients based on SARA score).

#### MRI data

##### Voxel-based morphometry

**Figure 6.**
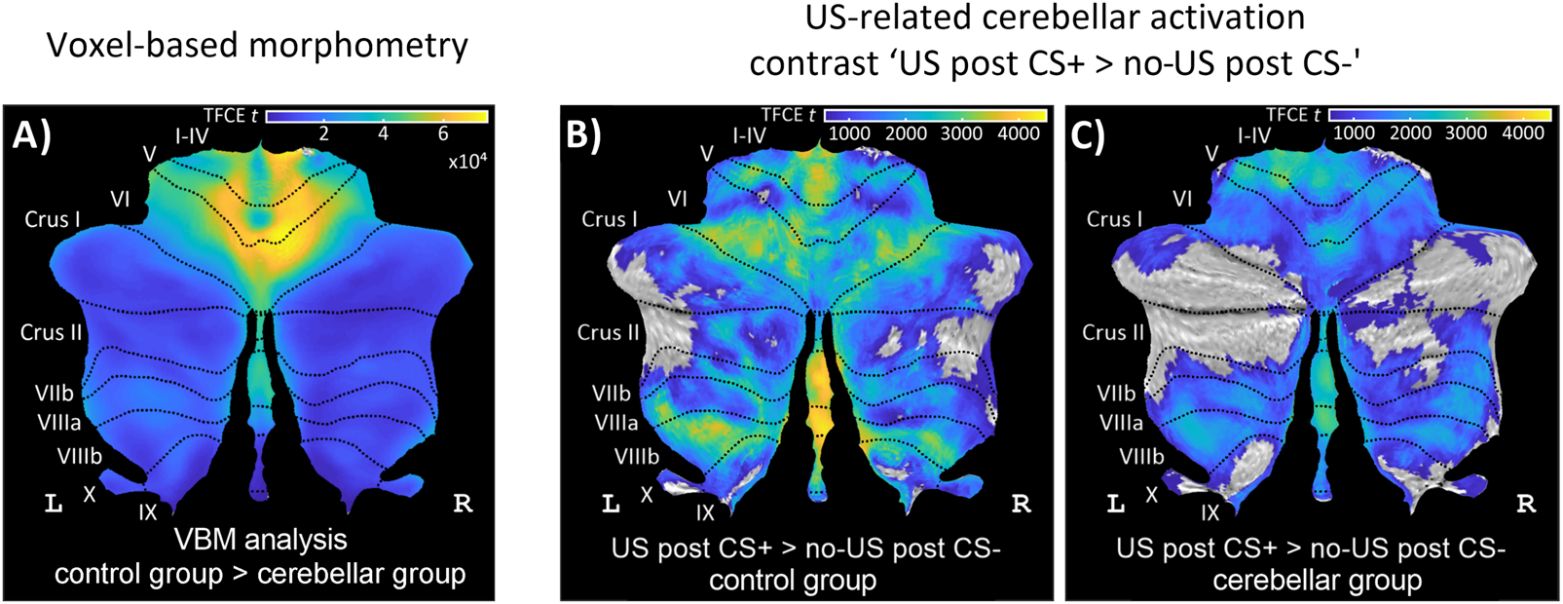
Results of voxel-based morphometry and cerebellar activation related to the presentation of the aversive US. ***A***, gray matter voxel-based morphometry (contrast “control group > cerebellar group”). US-related cerebellar activation (contrast “US post CS+ > no-US post CS−” during fear acquisition training) in (***B***) healthy controls and (**C**) cerebellar patients collapsed over early and late fear acquisition blocks. VBM group results and cerebellar activations are calculated using TFCE and FWE correction (*p* < 0.05) and in SUIT space projected on a cerebellar flatmap (Diedrichsen and Zotow, 2015). VBM, voxel-based morphometry; CS, conditioned stimulus; L, left; R, right; SUIT, spatially unbiased atlas template of the cerebellum; TFCE, threshold-free cluster enhancement; FWE, family-wise error rate; US, unconditioned stimulus. Additional details for VBM analysis are provided in Extended Data Table 6-1. Results of fMRI analysis are provided in Extended Data Table 6-2. For VBM analysis of patient subgroups, see Extended Data Figure 6-1.

VBM analysis revealed degeneration throughout the entire cerebellar cortex in the patient group. Using a two-sample *t* test, the control group > cerebellar group gray matter contrast (Fig. 6*A*) revealed a single large cluster (Extended Data Table 6-1). Degeneration was most prominent in the anterior lobe and lobule VI bilaterally (local maxima in right lobules I–IV, V, VI) but also in posterior parts of the vermis. The cerebellar group > control group gray matter contrast revealed no significant differences.

10.1523/ENEURO.0365-23.2023.f6-1 Figure 6-1 Gray matter voxel-based morphometry [contrast ‘control group > patient group’] for patient subgroups with **A)** spinocerebellar ataxia type 6 (SCA6, n = 6), **B)** sporadic adult-onset ataxia of unknown etiology (SAOA, n = 6) and **C)** autosomal dominant cerebellar ataxia type III (ADCA III, n = 5, SCA6 patients are not included in the ADCA III group). VBM group results are calculated using an uncorrected threshold p < 0.05 and in MNI space projected on a cerebellar flatmap using the SUIT toolbox (Diedrichsen and Zotow, 2015). VBM = voxel-based morphometry; L = left; R = right; SUIT = spatially unbiased atlas template of the cerebellum. Download Figure 6-1, TIF file.

10.1523/ENEURO.0365-23.2023.t6-1 Table 6-1 **Voxel-based morphometry results.** Gray matter clusters are reported which were significant after application of threshold-free cluster-enhancement (TFCE) at p < 0.05 FWE corrected level (second level t-test). One cluster is detected and displayed (isotropic voxel size: 0.6 mm). For this cluster, three local maxima are listed separated by at least 8 mm. Download Table 6-1, DOC file.

10.1523/ENEURO.0365-23.2023.t6-2 Table 6-2 Fear acquisition training, extinction training and recall. Activation clusters are reported which were significant after application of threshold-free cluster-enhancement (TFCE) at *p* < 0.05 FWE corrected level (*t*-tests). Displayed are all clusters ≥ 10 voxel (isotropic voxel size: 1.7 mm). In each cluster, up to three maxima are listed separated by at least 8 mm. CS+ = CS+E and CS+U trials were collapsed into the CS+. ncl. = nucleus, *p_FWE_* = family-wise error *p* value. Download Table 6-2, DOC file.

We performed additional VBM analysis in the three major subgroups of patients to ensure that the pattern of degeneration was not different. We found that patients with SCA6 (*n *= 6), ADCA III (*n* = 5), and SAOA (*n* = 6) showed very similar patterns of cerebellar degeneration; that is, vermal areas and the anterior parts of the cerebellum were most severely degenerated. VBM analysis of subgroups is shown in Extended Data Figure 6-1.

#### Functional MRI data

##### Cerebellar activation related to presentation of the aversive stimulus (US)

Cerebellar activations related to the presentation of the aversive stimulus (contrast “US post CS+ > no-US post CS−”) were observed within the cerebellar vermis and both cerebellar hemispheres (Fig. 6*B*,*C*; see also Extended Data Table 6-2). In both controls and cerebellar patients, US-related activations were found in the anterior lobe extending into lobule VI of the posterior lobe, lobule VIII in the posterior lobe, and the posterior vermis. Activations of Crus I and Crus II in the posterior lobe appeared to be more extended in controls compared with those in patients. Group comparison did not reveal significant differences. Of note, fMRI activations in patients overlapped with the areas in the cerebellar cortex in which cerebellar degeneration was most prominent (i.e., anterior lobe extending into lobule VI and cerebellar vermis; Fig. 6*A*).

##### Cerebellar activation in fear acquisition training

**Figure 7.**
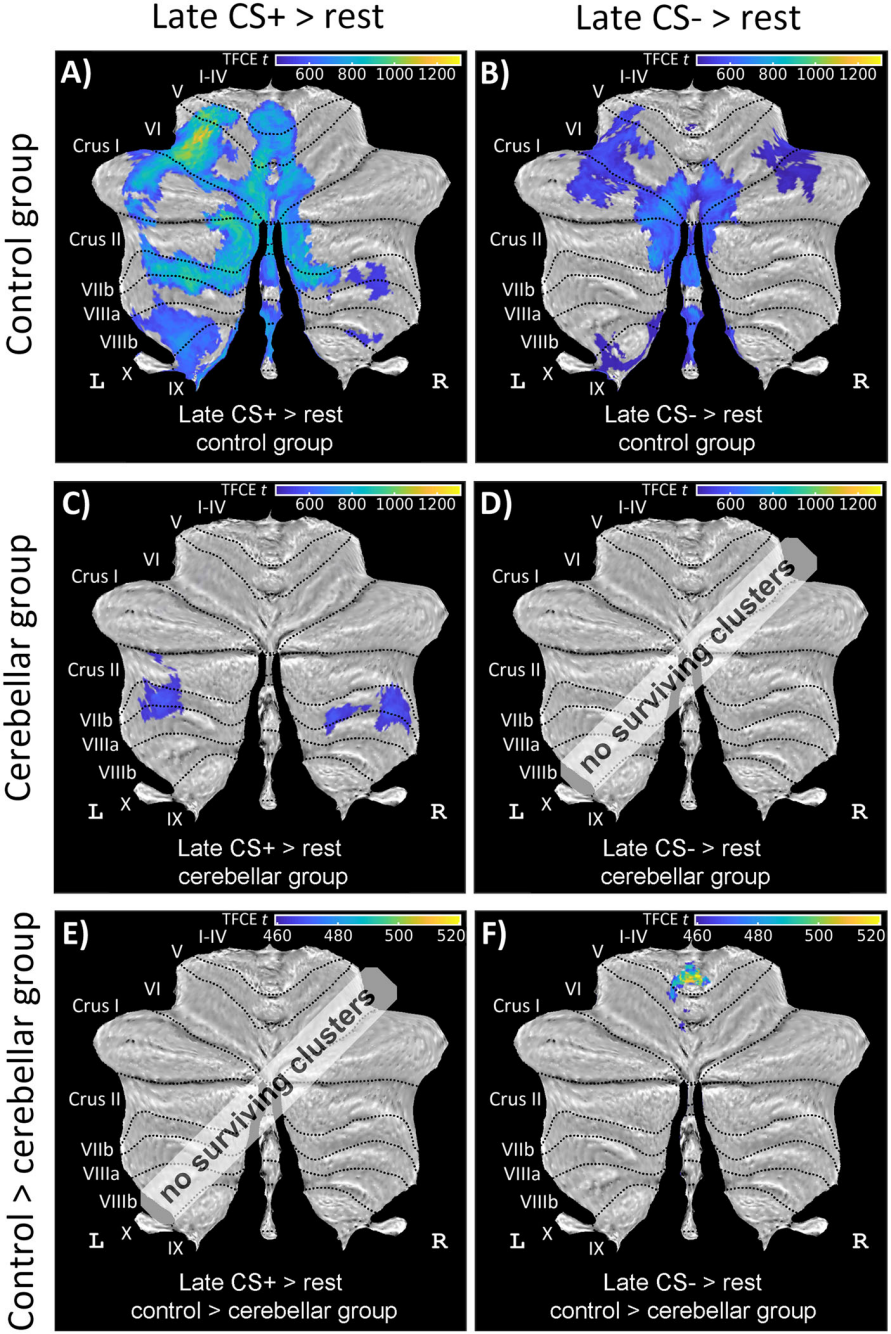
Cerebellar activation related to the CS+ and CS− during late fear acquisition training in healthy controls (top row), patients (middle row), and a comparison between controls and patients (bottom row). Cerebellar activations during the presentation of (***A***, ***C***) CS+ (contrast “CS+ > rest”), (***B***, ***D***) CS− (contrast “CS− > rest”) during late fear acquisition. ***E***, ***F***, In late acquisition CS− shows increased activation in comparison with early acquisition (contrast “CS−, late > early”). All contrasts are calculated using TFCE and FWE correction (*p* < 0.05) and presented in SUIT space projected on a cerebellar flatmap (Diedrichsen and Zotow, 2015). CS, conditioned stimulus; L, left; R, right; SUIT,  spatially unbiased atlas template of the cerebellum; TFCE, threshold-free cluster enhancement; FWE, family-wise error rate. No surviving clusters = no significant clusters ≥10 voxel (isotropic voxel size, 1.7 mm) after application of TFCE at *p* < 0.05 FWE corrected level. Results of fMRI analysis are provided in Extended Data Table 6-2. Group comparisons revealed significantly more cerebellar activations related to the CS− in controls compared with those in patients, while no significant group differences were found for the CS+. Note that TFCE correction takes values of neighboring voxels into account which is different to voxel-based correction. Despite the widespread activations in the control group, activations remain relatively weak and likely explain the absence of a significant group difference, despite clear differences based on (***A***) and (***C***).

*Activations related to the prediction of the US (in CS+ trials) no-US (in CS− trials)*. During early fear acquisition training, neither patients nor controls revealed significant cerebellar activations related to the CS+ (collapsing the CS+E and CS+U trials together into CS+, contrast “CS+ > rest”) or the CS− (contrast “CS− > rest”; Extended Data Table 6-2). During late fear acquisition training, controls revealed significant activations related to the CS+ in lateral and intermediate parts of lobules VI and Crus I bilaterally and the posterior vermis (Fig. 7*A*; see also Extended Data Table 6-2). In cerebellar patients, significant activations related to the CS+ were observed in lobule VIIb bilaterally (Fig. 7*C*). In controls, related to the CS−, significant bilateral activations were observed in lateral and intermediate parts of lobule VI and Crus I as well as lobule VII and VIII, primarily on the left, as well as vermis (Fig. 7*B*). Cerebellar patients revealed no significant activations related to the CS−. Overall, the activations appeared to be more pronounced in controls compared with those in patients. Group comparisons showed significantly more activations related to the CS− in the anterior vermis in controls compared with those in patients, while no significant group differences were found for the CS+ (Fig. 7*E*,*F*; Extended Data Table 6-2). Furthermore, no significant differences between stimulus-type events (CS+ vs CS−) were found either in patients or in controls.

**Figure 8.**
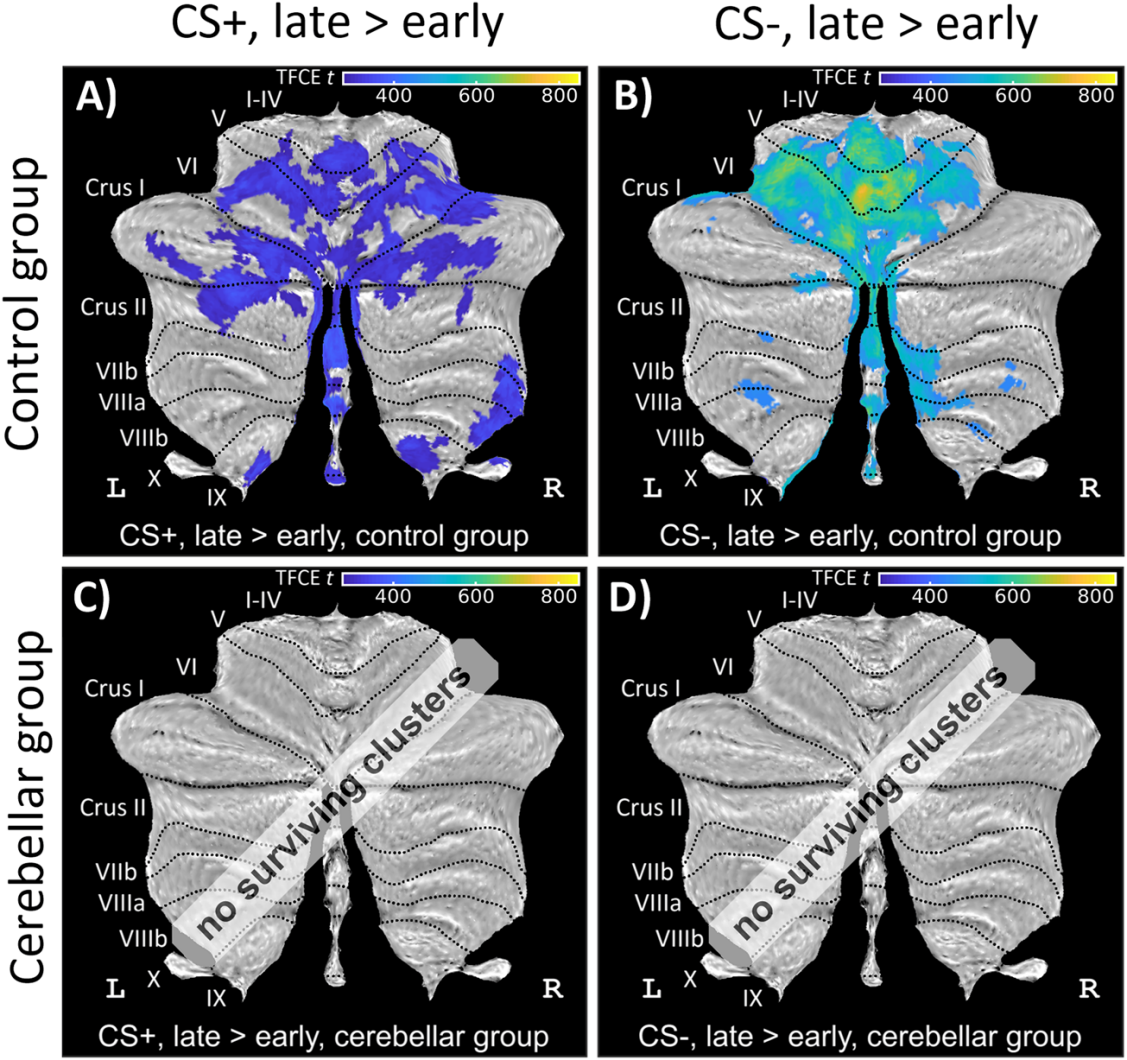
Cerebellar activation related to the CS during fear acquisition training (contrast “CS+ late > early”; left column and contrast “CS− late > early”; right column) in (***A***, ***B*)** healthy controls and (***C***, ***D***) cerebellar patients in SUIT space projected on a cerebellar flatmap (Diedrichsen and Zotow, 2015). All contrasts are calculated using TFCE and FWE correction (*p* < 0.05). CS, conditioned stimulus; L, left; R, right; SUIT, spatially unbiased atlas template of the cerebellum; TFCE, threshold-free cluster enhancement; FWE, family-wise error rate. No surviving clusters = no significant clusters ≥10 voxel (isotropic voxel size, 1.7 mm) after application of TFCE at *p* < 0.05 FWE corrected level. Results of fMRI analysis are provided in Extended Data Table 6-2. Controls exhibited significantly higher cerebellar activations related to the presentation of both CS+ and CS− during late fear acquisition training compared with early training. Patients did not show any cerebellar activations.

In controls, cerebellar activations related to the CS+ and CS− presentation were significantly higher during late compared with those during early fear acquisition training (contrasts “CS+ late > early” and “CS− late > early”). These activations included clusters in the anterior lobe, lobule VI extending into Crus I and Crus II, and the vermis bilaterally (Fig. 8*A*,*B*; Extended Data Table 6-2). No cerebellar activations were detected in patients. Between-group differences were not significant.

**Figure 9.**
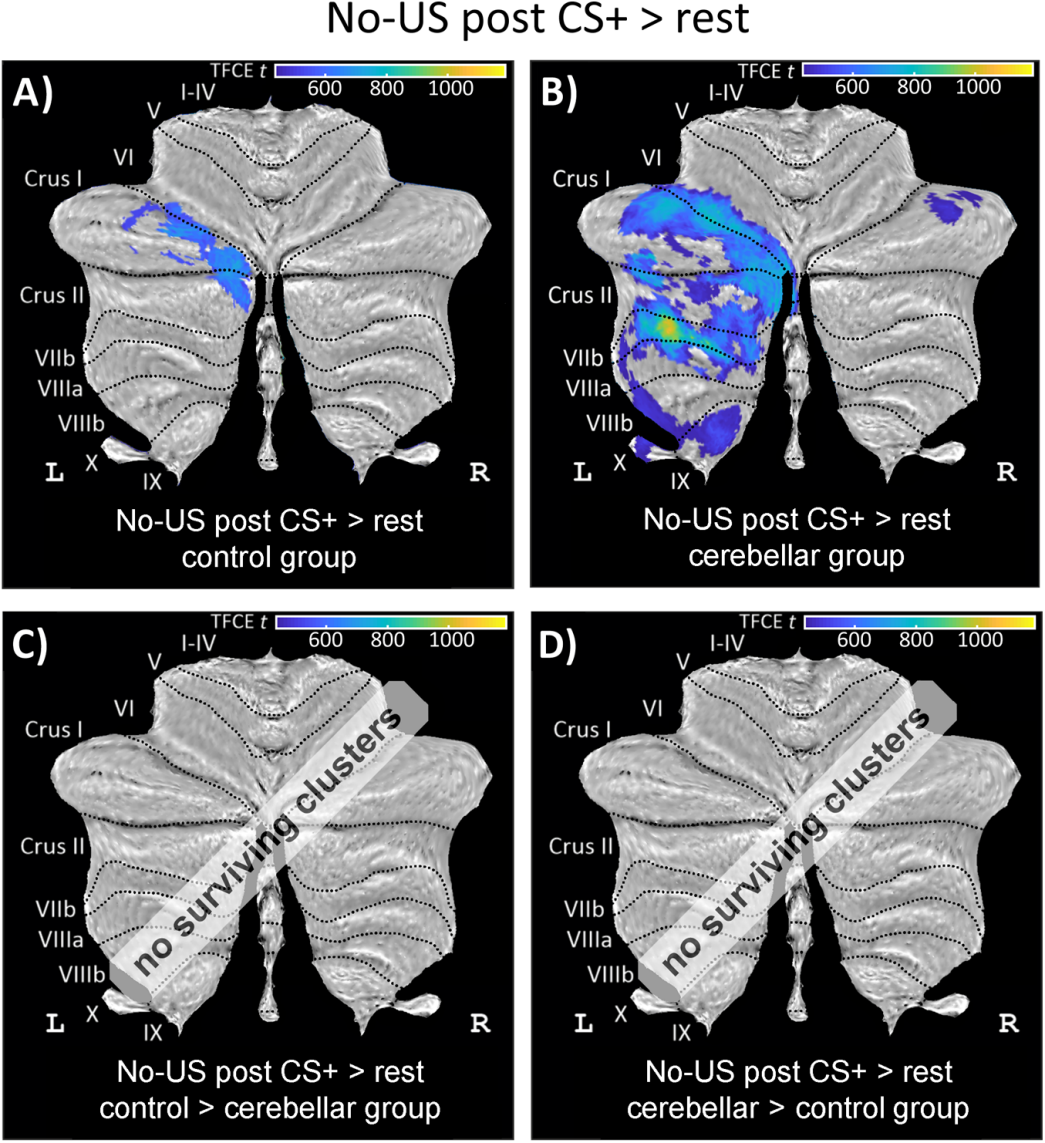
Cerebellar activation related to the omission of the aversive US during acquisition training (contrast “no-US post CS+ > rest”) in (***A***) healthy controls, (***B***) cerebellar patients, and (***C***, ***D***) comparison between controls and patients in SUIT space projected on a cerebellar flatmap (Diedrichsen and Zotow, 2015). All contrasts are calculated using TFCE and FWE correction (*p* < 0.05). CS, conditioned stimulus; L, left; R, right; SUIT, spatially unbiased atlas template of the cerebellum; TFCE, threshold-free cluster enhancement; FWE, family-wise error rate; US, unconditioned stimulus. No surviving clusters = no significant clusters ≥10 voxel (isotropic voxel size, 1.7 mm) after application of TFCE at *p* < 0.05 FWE corrected level. Results of fMRI analysis are provided in Extended Data Table 6-2. Cerebellar activations were more prominent in patients compared with those in controls with no significant difference between groups.

*Activations related to the unexpected omission of the US in CS+ and CS− trials*. In both cerebellar patients and controls, significant cerebellar activations were observed at the time when the aversive US was expected but did not occur in unreinforced CS+ trials (contrast “no-US post CS+ > rest”). In controls, activations were observed in left Crus I with some extension into Crus II. In patients, activations related to the omission of the aversive US were observed in left lobules VI, Crus I, and Crus II extending into lobules VII–VIII (Fig. 9*A*,*B*; Extended Data Table 6-2). Overall, activations were more prominent in patients compared with those in controls. Between-group differences, however, were not significant. In CS− trials, at the time the US was presented in CS+ trials, no cerebellar activation was observed except a small cluster in left lobule VIIb in controls. No significant differences between stimulus-type events (“no-US post CS+ > no-US post CS−”) were found either in patients or in controls.

##### Cerebellar activation in fear extinction training

*Activations related to the prediction of the US (in CS+E trials)/no-US (in CS− trials)*. During both early and late fear extinction training, no voxels showed significant activation related to CS presentation (contrasts “CS+E > rest”, “CS− > rest”) or when comparing stimulus-type events (contrasts “CS+E > CS−”, “CS− > CS+E”) in cerebellar patients or in controls.

**Figure 10.**
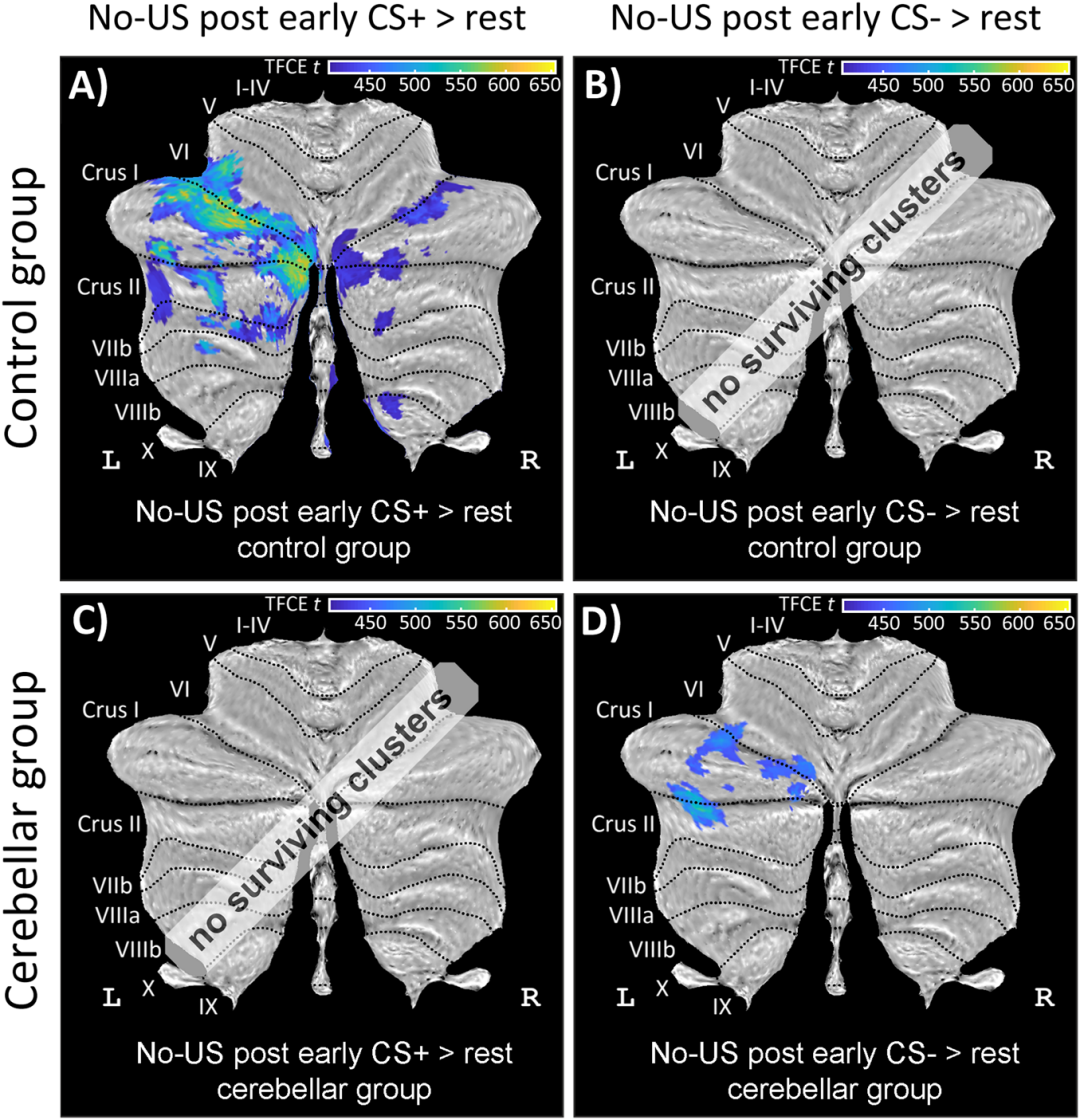
Cerebellar activation related to the omission of the aversive US during early extinction training (contrasts “no-US post CS+ > rest” and “no-US post CS− > rest”) in healthy controls (top row) and cerebellar patients (bottom row) in SUIT space projected on a cerebellar flatmap (Diedrichsen and Zotow, 2015). All contrasts are calculated using TFCE and FWE correction (*p* < 0.05). CS, conditioned stimulus; L, left; R, right; SUIT, spatially unbiased atlas template of the cerebellum; TFCE, threshold-free cluster enhancement; FWE, family-wise error rate; US, unconditioned stimulus. No surviving clusters = no significant clusters ≥10 voxel (isotropic voxel size, 1.7 mm) after application of TFCE at *p* < 0.05 FWE corrected level. Results of fMRI analysis are provided in Extended Data Table 6-2. During early extinction training, controls exhibited significant no-US–related cerebellar activations in CS+ trials, whereas cerebellar patients showed significant no-US–related activations toward CS− trials.

*Activations related to the unexpected omission of the US in CS+E and CS− trials*. During early extinction, training controls exhibited significant no-US–related activations in CS+ trials (i.e., at the time the US was presented in paired acquisition trials but did not occur in extinction trials; contrast “no-US post CS+ > rest”) in lobules VI, Crus I, Crus II extending into lobule VII of the left cerebellar hemisphere, as well as right Crus I and Crus II. No activations were observed in CS− trials (contrast “no-US post CS− > rest”; Fig. 10*A*,*B*; Extended Data Table 6-2). In patients, no significant activations were detected in CS+ trials, but significant no-US–related activations were detected in CS− trials (contrast “no-US post CS− > rest”) in lobules VI, Crus I, and Crus II of the left cerebellar hemisphere (Fig. 10*C*,*D*; Extended Data Table 6-2). No significant between-group differences were found.

During late extinction, no significant cerebellar activation was observed at the time of the omission of the US in both patients and controls.

##### Cerebellar activation in recall

Because SCR amplitude and questionnaires did not reveal significant differences comparing CS+E and CS+U, decision was made to analyze CS+E and CS+U together, similar to the fear acquisition training.

**Figure 11.**
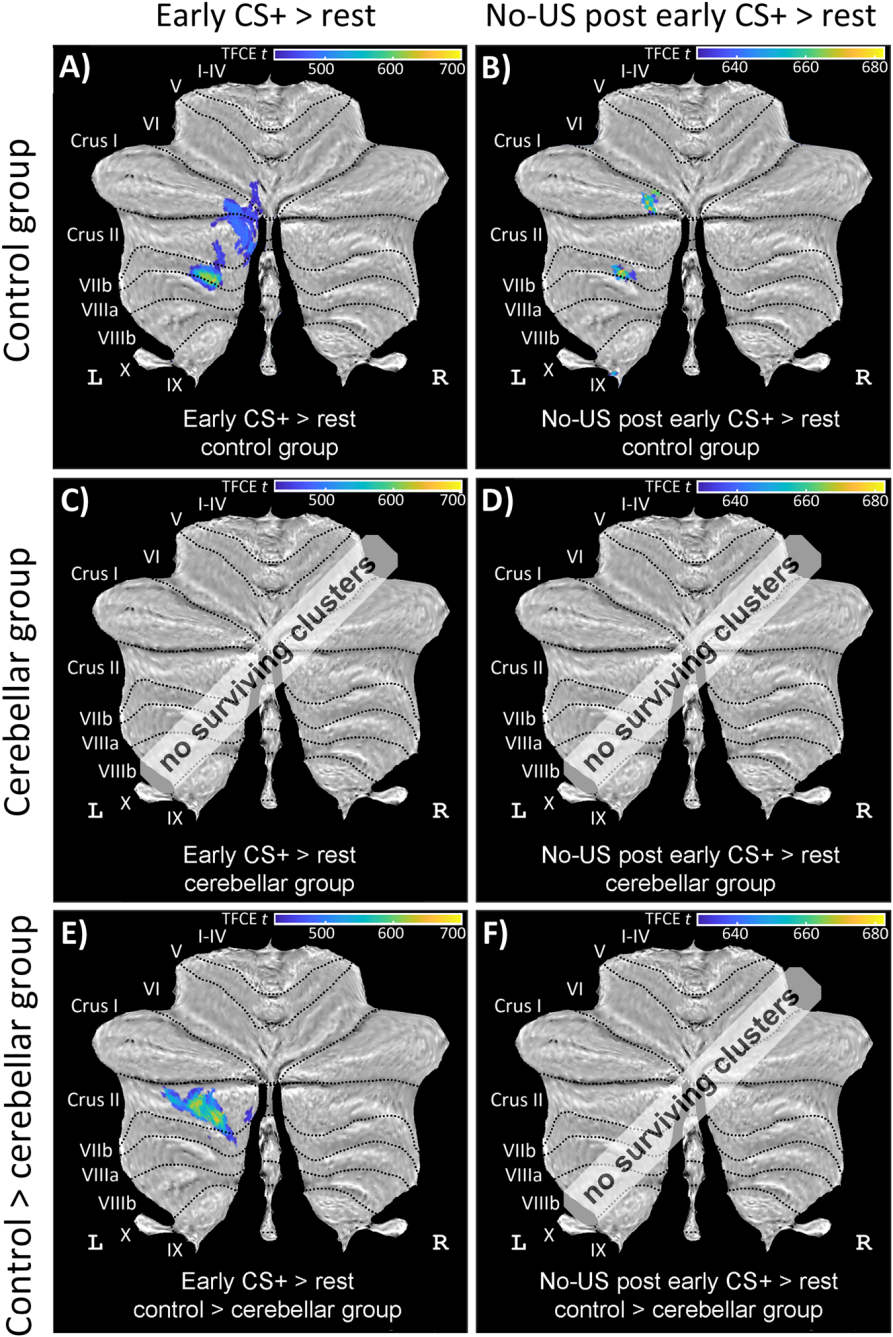
Cerebellar activation related to the CS and omission of the aversive US during early recall (contrasts “CS+ > rest” and “no-US post early CS+ > rest”) in healthy controls (top row), cerebellar patients (middle row), and a comparison between controls and patients (bottom row) in SUIT space projected on a cerebellar flatmap (Diedrichsen and Zotow, 2015). All contrasts are calculated using TFCE and FWE correction (*p* < 0.05). CS, conditioned stimulus; L, left; R, right; SUIT, spatially unbiased atlas template of the cerebellum; TFCE, threshold-free cluster enhancement; FWE, family-wise error rate; US, unconditioned stimulus. No surviving clusters = no significant clusters ≥10 voxel (isotropic voxel size, 1.7 mm) after application of TFCE at *p* < 0.05 FWE corrected level. Results of fMRI analysis are provided in Extended Data Table 6-2. Controls exhibited significant cerebellar activations related to the CS+ presentation and significant cerebellar activations related to the omission of the US in CS+ trials. Group comparisons revealed significantly stronger activations related to the CS+ presentation in controls compared with those in patients.

*Activations related to the prediction of the US (in CS+ trials)/no-US (in CS− trials)*. During early recall, significant cerebellar activations were found related to the CS+ presentation in the left intermediate posterior cerebellar hemisphere primarily within cerebellar lobules Crus I, Crus II, and VIIb in controls (Fig. 11*A*; Extended Data Table 6-2). In patients, no significant cerebellar activation was observed related to the CS+ in early recall (Fig. 11*C*; Extended Data Table 6-2). Group comparisons revealed significantly more activations related to the CS+ in left Crus II with some extension to lobule VII in controls compared with those in patients (Fig. 11*E*; Extended Data Table 6-2). No significant activations were revealed related to the CS− neither in patients nor controls. During late recall, no significant cerebellar activation was observed in both patients and controls.

*Activations related to the unexpected omission of the US in CS+ trials*. During early recall, significant cerebellar activations were detected in controls related to the omission of the US in CS+ trials in left Crus I and lobule VIIb (contrast “no-US post CS+ > rest”; Fig. 11*B*; Extended Data Table 6-2), but not in CS− trials (“no-US post CS− > rest”). In patients, no voxels showed significant activation related to the omission of the US (contrasts “no-US post CS+ > rest” and “no-US post CS− > rest”, Fig. 11*D*; Extended Data Table 6-2). No significant group differences were found related to the omission of the US after CS+ and CS− (Fig. 11*F*; Extended Data Table 6-2).

During late recall, no significant cerebellar activation was observed at the time of the omission of the US in neither patients nor controls.

### Animal study

**Figure 12.**
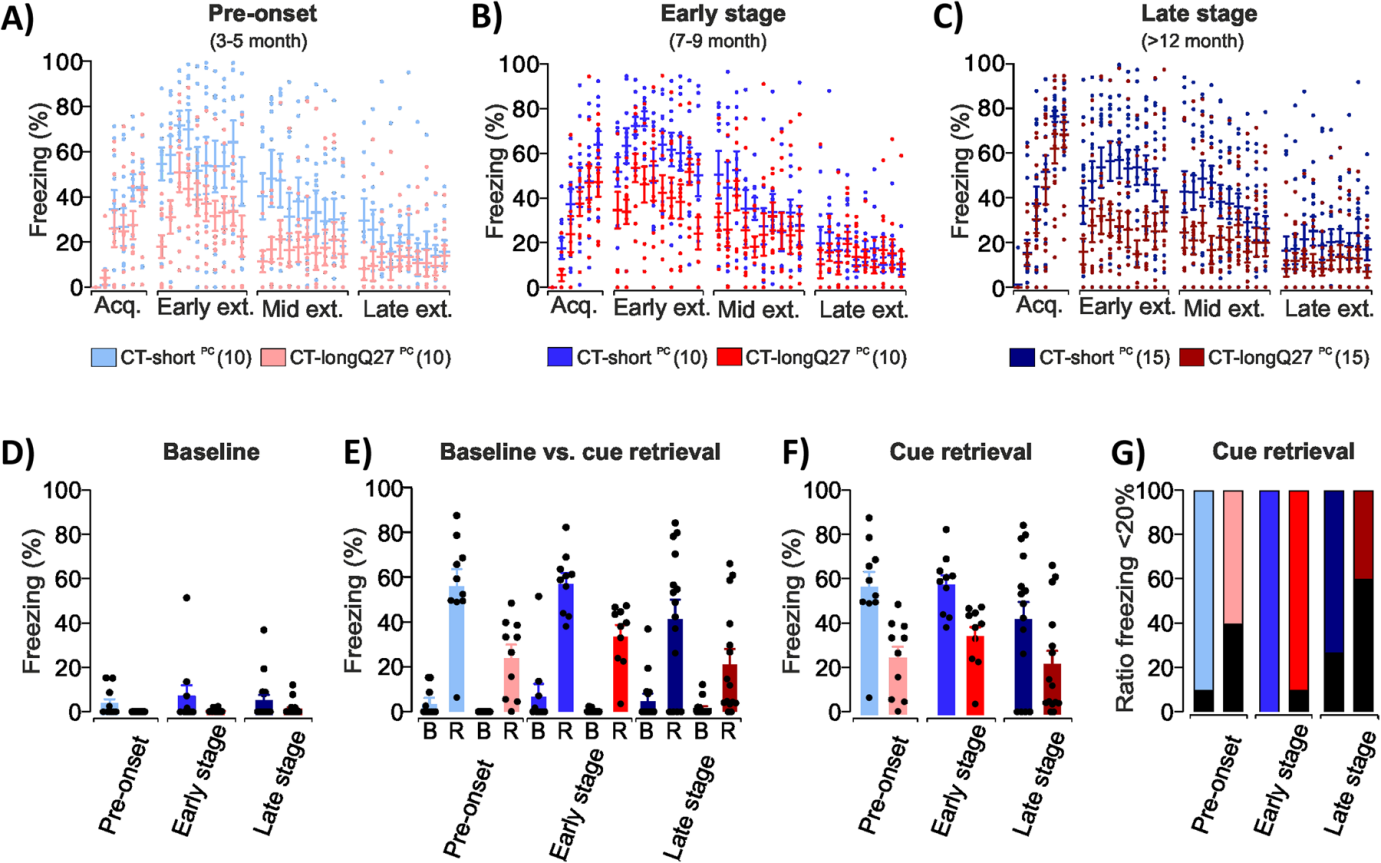
Results in SCA6 mouse model (CT-longQ27^PC^). Deficits are demonstrated in early fear extinction training (i.e., during retrieval) in all stages of the disease. ***A***, Pre-onset CT-short^PC^ (light blue) and CT-longQ27^PC^ (light red) mice were analyzed for freezing behavior during the 30 s conditioning stimulus (CS) in fear acquisition (acq.) and extinction training (ext.). Pre-onset CT-longQ27^PC^ mice displayed lower freezing levels during early extinction. ***B***, Early stage CT-longQ27^PC^ (red) mice displayed reduced freezing levels during early extinction in comparison with CT-short^PC^ (blue) mice. ***C***, Freezing behavior for late stage CT-short^PC^ (dark blue) and CT-longQ27^PC^ (dark red) mice during the CSs of fear acquisition and extinction training revealed no differences in freezing behavior between mouse lines. ***D***, Baseline freezing as a sign of generalized fear revealed that CT-longQ27^PC^ mice did not display significantly different freezing levels in comparison with CT-short^PC^ mice. ***E***, Comparison of baseline (B) and retrieval (R) freezing of each group revealed that all groups display significantly higher freezing during the CS presentation concerning their previous baseline freezing. ***F***, Freezing behavior during cue retrieval, which is defined as the percentage of freezing to the first two CS presentations during early extinction, was analyzed to show fear-related learning to the cue and revealed that CT-longQ27^PC^ mice display lower freezing levels in comparison with CT-short^PC^ mice during the pre-onset and early stage of the disease. ***G***, Percentage of mice per group displaying freezing responses below 20% (black) and above 20% (corresponding group color) during cue retrieval. ***A–F***, Mean ± SEM with individual animals as single points. The number of animals is indicated in parentheses behind the group. Statistical findings are summarized in Extended Data Tables 12-1, 12-2, and 12-3.

To determine whether the progressive SCA6 disease affects fear behavior, we tested CT-longQ27^PC^ and CT-short^PC^ as control mice (Fig. 2*A*) at different ages (pre-onset, early stage, and late stage) of the SCA6 disease in a single-cue fear conditioning paradigm. A tone (US) was combined with an aversive stimulus six times during fear acquisition training to achieve fear conditioning to the tone. Extinction learning occurred in three sessions, early, mid, and late extinction training where each session consisted of 10 CS. Extinction training took place on 3 consecutive days (Fig. 2*B*). At all stages (pre-onset, early stage, and late stage) of the SCA6 disease, CT-longQ27^PC^ and CT-short^PC^ mice demonstrated increasing freezing responses during fear acquisition training (nonparametric two-way ANOVA-type statistic for repeated measures; all *p* values <0.001; Fig. 12*A–C*; Extended Data Table 12-1) with no differences between genotypes. Early extinction training revealed that CT-longQ27^PC^ mice exhibited overall lower freezing levels than CT-short^PC^ mice at all disease stages (nonparametric two-way ANOVA-type statistic for repeated measures; all *p* values ≤ 0.012), with significant decreases of fear responses within the early extinction session (nonparametric two-way ANOVA-type statistic for repeated measures; all *p* values ≤ 0.004). Decreasing fear behavior was further observed during mid extinction learning within the early and late stage groups (nonparametric two-way ANOVA-type statistic for repeated measures, all *p* values ≤ 0.01), but not in the pre-onset group which did not show a significant change in fear behavior after the early extinction session. Additionally, freezing levels from CT-longQ27^PC^ mice were significantly lower at the late disease stage when compared with CT-short^PC^ mice (nonparametric two-way ANOVA-type statistic for repeated measures; *F*_(1)_ = 4.07; *p *= 0.044), while there was no statistical difference between genotypes for the pre-onset and early stage mice (nonparametric two-way ANOVA-type statistic for repeated measures; all *p* values ≥0.186). During late extinction, no changes in freezing behavior and no differences between the genotypes were evident (nonparametric two-way ANOVA-type statistic for repeated measures; all *p* values for genotype ≥0.075; for trial ≥0.181). Furthermore, baseline (Fig. 12*D*) freezing, defined as fear behavior in the extinction context without any CS presentation, and retrieval (Fig. 12*F*), which reflects the first two CSs during early extinction, were analyzed and compared with each other (Fig. 12*E* and Extended Data Tables 12-2 and 12-3; nonparametric two-way ANOVA-type statistic for repeated measures and respective post hoc analysis). Post hoc analysis showed significantly higher freezing responses during baseline for the pre-onset CT-short^PC^ mice when compared with CT-longQ27^PC^ (post hoc *p *= 0.028). Furthermore, during recall, pre-onset and early stage CT-longQ27^PC^ mice exhibited lower freezing levels compared with their respective controls (all *p* values ≥0.001), while the late stage group did not. Within-group comparisons between baseline and recall demonstrated significantly more fear to the tone than during baseline in all groups (all *p* values ≥0.001), indicating that fear memory was acquired. To gain further insight into the difference in fear retrieval between CT-longQ27^PC^ and control mice, we analyzed the proportion of animals freezing <20% and >20% (Fig. 12*G*). CT-longQ27^PC^ mice were more likely to express low freezing levels, especially at the late disease stage compared with their age-matched controls. Altogether, these results indicate that CT-longQ27^PC^ mice display decreased fear responses to auditory cue conditioning during retrieval and early extinction.

10.1523/ENEURO.0365-23.2023.t12-1 Table 12-1 Results of the non-parametric two-way repeated measures ANOVA for freezing behavior between CT-short^PC^ and CT-longQ27^PC^ mice groups. Download Table 12-1, DOC file.

10.1523/ENEURO.0365-23.2023.t12-2 Table 12-2 Results of the non-parametric two-way ANOVA-type statistic for freezing behavior between genotype and trial during baseline and retrieval. Download Table 12-2, DOC file.

10.1523/ENEURO.0365-23.2023.t12-3 Table 12-3 *Post hoc* comparisons for freezing behavior between genotype and trial during baseline and retrieval (least squares means test). Download Table 12-3, DOC file.

## Discussion

Cerebellar cortical degeneration led to mild deficits in fear learning in both humans and mice. Among cerebellar patients, there were some indications of slowed acquisition and delayed consolidation of learned fear as well as slowed extinction. In mutant mice, retrieval of learned fear was reduced. In cerebellar patients, mild behavioral abnormalities were accompanied by changes in fMRI signal in the cerebellar cortex, suggesting alterations in inputs related to the prediction and unexpected omission of the aversive stimulus. A more detailed discussion of these findings is provided below.

### Behavioral data

Both patients with cerebellar cortical degeneration and controls showed significant differential fear conditioning, based on skin conductance responses and questionnaires. SCRs and fear ratings were higher toward the CS+s compared with CS− toward the end of fear acquisition training. Controls, however, showed higher SCR incidences in response to the CS+ compared with the CS− already during early acquisition training, whereas this pattern only emerged in cerebellar patients during late acquisition training. In addition, patients required significantly more time than controls to recognize the CS/US contingencies. Findings suggest slower learning of fear associations in patients. Given that contingency awareness is linked to working memory processes (Dawson and Furedy, 1976), deficits in working memory may also contribute (Schmahmann and Sherman, 1998; Ravizza et al., 2005).

Furthermore, after the first session of extinction training, controls reported significantly more negative valence, higher arousal, and fear toward the CS+, along with higher US expectancy ratings after the CS+ compared with those after CS−. This aligns with past human studies reporting that fear-related SCRs extinguish during extinction training, whereas differential responses in questionnaires persist (Vansteenwegen et al., 2006; Blechert et al., 2008; Inoue et al., 2020; Batsikadze et al., 2022). In contrast, responses from cerebellar patients did not significantly differ in these questionnaires when comparing CS+ and CS− postextinction training. In our human study, fear acquisition and the first session of extinction training were performed within the same session on day 1. In the second extinction session on day 2 (“recall“ phase), both groups showed spontaneous recovery of the learned associations from acquisition training in SCRs and questionnaires. This suggests that more time was required to achieve complete consolidation of learned fear associations in cerebellar patients. Finally, throughout the second extinction session on day 2, SCR incidence was significantly higher in CS+U trials compared with CS− trials in cerebellar patients, but not in controls. The CS+U was not extinguished on day 1, and findings indicate that extinction learning was slowed in cerebellar patients.

Similar to our findings in patients, mice with SCA6 were able to learn the association between CS and shock: both SCA6 and control mice showed increasing freezing responses toward the CS during fear acquisition training, irrespective of the disease stage. Findings are consistent with results from other SCA animal models (Huynh et al., 2009; Asher et al., 2020). Both pre-onset and early stage SCA6 mice exhibited significantly reduced retrieval of the learned fear association during initial extinction trials. Reduced retrieval could not be explained by reduced baseline freezing levels. These differences may be attributed to an altered fear consolidation or processing of the CS–US association. In either scenario, these alterations could lead to modifications in fear memory processing which manifest during retrieval. These results are in good accordance with previous findings of reduced retrieval in mouse models of other types of cerebellar degeneration (Huynh et al., 2009; Asher et al., 2020). In addition, recent research manipulating the interaction between the fastigial nuclei (FN) and the periaqueductal gray (PAG) in wild-type mice (Frontera et al., 2020) supports our findings. Opto- and chemogenetic manipulations of the direct FN-PAG pathway during fear acquisition training resulted in alterations in retrieval and extinction learning without abnormalities in freezing behavior during acquisition training. Likewise, optogenetic manipulation of cerebellar nuclei neurons projecting to the lateral parabrachial nucleus resulted in altered fear retrieval (Hwang et al., 2023).

Despite reduced retrieval, and unlike patients, extinction learning in all three sessions was not different between SCA6 mice and age-matched controls. Extinction learning was age dependent and declined in older mice, but independent of the genotype. Direct comparison between patients and mice, however, is limited because of differences in fear conditioning paradigms (differential vs single cue conditioning) and outcome parameters (SCRs and questionnaires vs freezing responses; Doubliez et al., 2023).

Overall, behavioral abnormalities were mild both in humans and mice with cerebellar cortical degeneration. This differs from findings in motor associative learning, such as eyeblink conditioning, but also locomotor (e.g., split-belt walking; Statton et al., 2018) and reach adaptation (Rabe et al., 2009; Donchin et al., 2012; Burciu et al., 2014; Honda et al., 2018). Previous studies reported significant reductions of conditioned eyeblink acquisition in patients and mouse models with cerebellar cortical degeneration, including SCA6, regardless of the disease stage (Topka et al., 1993; Gerwig et al., 2005; Timmann et al., 2005; Mark et al., 2015; van Gaalen et al., 2019). Furthermore, the cerebellar circuitry underlying fear conditioning and eyeblink conditioning may be distinct and differentially affected in cerebellar cortical degeneration. For example, whereas long-term depression (LTD) at the parallel fiber→Purkinje cell synapse is known to contribute to eyeblink conditioning (Boele et al., 2018), long-term potentiation (LTP) has been shown to be involved in fear conditioning (Sacchetti et al., 2005), with climbing fiber input being involved in LTD but not LTP. Furthermore, in contrast to eyeblink conditioning, the contribution of the cerebellar cortex may be less essential to fear conditioning. Fear conditioning involves an extended neural network including the amygdala (Milad et al., 2007; Linnman et al., 2012; Fullana et al., 2016). The remaining fear neural network might compensate for cerebellar deficits particularly in slowly progressive disease. Surgical lesions of the vermis and fastigial nuclei (Chambers and Sprague, 1955; Sprague and Chambers, 1959; Supple et al., 1987) may reveal more prominent deficits in fear conditioning because they result in more complete and/or acute dysfunction.

In sum, cerebellar cortical degeneration led to mild abnormalities in fear learning in humans and mice manifesting to large extent postacquisition training.

### Cerebellar fMRI signals in fear conditioning in patients with cerebellar degeneration

Patterns of fear learning-related cerebellar cortical activations were different between cerebellar patients and controls, although direct comparisons between groups did not consistently reach statistical significance. In brief, during acquisition training, cerebellar cortical activation related to the prediction of the US was less pronounced in patients compared with that in controls. Likewise, in recall, cerebellar cortical fMRI activation related to the prediction of the US in CS+ trials was significantly higher in controls than that in cerebellar patients. Furthermore, fMRI activation related to the unexpected omission of the US in unreinforced CS+ trials showed differences between controls and patients. During acquisition training in controls, the activated region closely matched the maximum activation in Crus I observed in previous studies (Ernst et al., 2019). In patients, the area was more extended, and the maximum was observed in lobule VIIb (Fig. 9*A*,*B*). During extinction training and recall, activations related to the unexpected omission toward the CS+ were present in controls, but not in patients.

Notably, fMRI signal of the cerebellar cortex has been related to input to the cerebellum, mainly mossy fiber input (Diedrichsen et al., 2010). Consequently, the present findings suggest that input related to the prediction and unexpected omission of the US is altered in cerebellar degeneration, although the exact nature of these alterations remains to be determined. Among other factors, reward and punishment signals may potentially play a role. Recent animal data suggests that the cerebellar cortex receives afferent input of the presentation and the prediction of rewards and reward prediction errors (Wagner et al., 2017; Hull, 2020, for review; Kostadinov and Häusser, 2022, for review). While studies have been conducted in less detail, they indicate that cerebellar granule cells also receive information about the aversiveness of a stimulus (Shuster et al., 2021).

Prediction errors are thought to drive fear learning (Rescorla and Wagner, 1972). The unexpected omission of the US is considered rewarding, and the reward prediction error is thought to drive extinction learning (Kalisch et al., 2019). Consequently, reduced or altered input of reward prediction error signals to the cerebellar cortex related to the unexpected omission of the US may contribute to slowed extinction learning. Similarly, reduced prediction error signal related to initial presentation of the US during acquisition training may contribute to decelerated fear learning. This, however, could not be tested because of the US-related widespread activation of the cerebellar cortex. The fMRI signals related to unexpected omissions, however, do not necessarily represent the valence of a stimulus; they may also represent a surprise signal or increased attention to an unexpected event (Lachnit et al., 2008; Fouragnan et al., 2018).

Importantly, the general ability of cerebellar patients to activate the cerebellar cortex appeared to be intact, and the observed differences cannot be explained by cerebellar atrophy. Cerebellar activation related to the aversive US, which is a mix of sensory, pain, and valence inputs, did not significantly differ between patients and controls, despite significant cerebellar cortical atrophy. Activation related to the aversive stimulus was most pronounced in the anterior cerebellum with extension into lobule VIIa, lobule VIII of the posterior lobe, and the vermis. This pattern closely matched the cerebellar areas related to aversive electrical stimulation reported in other studies (Dimitrova et al., 2003; Michelle Welman et al., 2018; Ernst et al., 2019). Of note, the regions with most prominent activations in the anterior lobe overlapped with the areas showing most pronounced cerebellar atrophy. At first sight, this may appear counterintuitive. However, fMRI signals primarily reflect synaptic afferent input to the cerebellar cortex (Logothetis et al., 2001). Pure cerebellar cortical degeneration is thought to result from Purkinje cell degeneration and subsequent loss of the dendritic tree in the molecular layer, leading to the observed cerebellar cortical atrophy in MRI scans (Koeppen, 2005, 2018). On the other hand, the granule cell layer is generally considered to remain largely intact. As mentioned above, cerebellar cortical fMRI signals predominantly reflect the mossy fiber input (Diedrichsen et al., 2010), which appear to be preserved within our patient group, based on the data related to the aversive US. Therefore, any alterations in cerebellar cortical fMRI signal in patients likely reflect changes in afferent input and cannot simply be explained by the extent of cerebellar atrophy.

Future studies will be of interest to investigate the exact nature of altered input signals to the cerebellar cortex related to the prediction and unexpected omission of the (aversive) US. Similarly, it will be of interest to explore the alterations of cerebellar output signals (i.e., of the cerebellar nuclei) in cerebellar disease. The fMRI signal at the cerebellar nuclei level was not sufficient to address this question in the current dataset.

### Limitations

A direct comparison between the human study and the mouse study is limited because the human study included participants, not exclusively patients with SCA6, and did not allow a comparison across different disease stages.

In line with a previous 7 T fMRI study on young and healthy participants (Batsikadze et al., 2022), our study found no significant differences between cerebellar activations related to the CS+s and CS−. Both studies closely followed the experimental paradigm introduced by Milad et al. (2007), where fear acquisition and extinction training took place in two distinct contexts represented by pictures of two different office rooms. Cerebellar activation observed in these studies may represent the complex association among the cue, context, and the US, potentially concealing differential activations observed in previous studies using a consistent neutral context throughout (Ernst et al., 2019).

Furthermore, contrary to our expectations, we did not find a significant difference between the CS+E and CS+U during early recall. It was expected that the extinguished CS+ (CS+E) would result in reduced SCR/questionnaire responses during early recall compared with the unextinguished CS+ (CS+U). This, however, was not the case. We observed spontaneous recovery to the CS+E which was not different to the reaction to the CS+U. Unlike the original study by Milad et al. (2007), we decided to present the CS+E and CS+U in a randomized fashion in acquisition training phase, rather than in separate blocks. This may have hampered a clear differentiation between the two CS+. While the age of our study population may be a factor, it is worth noting that a recent behavioral study in young and healthy participants employing a similar paradigm (Albayrak et al., 2023) also reported no differentiation between the CS+E and CS+U in early recall.

### Conclusions

In sum, cerebellar cortical degeneration resulted in mild abnormalities in the acquisition of learned fear responses in both humans and mice. Patients demonstrated slower acquisition and extinction learning, accompanied with diminished consolidation of learned fear. The latter aligns with the reduced retrieval observed in SCA6 mice. Notably, the fMRI findings in cerebellar patients imply that input associated to the prediction of the US is reduced and input related to the unexpected omission of the US is altered. Given that the cerebellar fMRI primarily reflects mossy fiber input, the changes in fMRI signal suggest that input signals to the cerebellar cortex related to the prediction and unexpected omission of the (aversive) US are altered in cerebellar cortical degeneration. Future research is warranted to investigate the exact nature of these altered input signals related to fear learning.

## Synthesis

Reviewing Editor: David Schoppik, New York University Decisions are customarily a result of the Reviewing Editor and the peer reviewers coming together and discussing their recommendations until a consensus is reached. When revisions are invited, a fact-based synthesis statement explaining their decision and outlining what is needed to prepare a revision will be listed below. The following reviewer(s) agreed to reveal their identity: Marija Cvetanovic.

Major comments:

1. Do the mice they used indeed show degeneration? If yes, please describe briefly, not just by citing previous paper, the degree of cerebellar cortical degeneration. If not, their descriptions in Abstract and elsewhere should be carefully modified. For example, in Page 4 line 80, we recommend "performed using a transgenic mouse model of SCA6 which presents cerebellar cortical degenerations in humans."

2. Authors combine data from five different cerebellar ataxias and different stages of disease. Yet different SCAs may have dysfunction/pathology in different regions of the cerebellum, and later stages of disease also have worse pathology. Could authors please analyze the FC and MRI data per different stage of disease (e.g. per SARA score or disease duration) and for each type of ataxia? Further, we wondered if the manuscript could assay the degree of ataxia severity relative to the fear responses in SCA6 subjects (n=6, Table 1). Finally, the manuscript would be stronger for detailing any points consistent with SCA6 patients and transgenic mice?

3. It is intriguing to know that fear-conditioning was not dramatically affected compared to these motor learning capabilities in ataxia patients. If the authors view is correct, it may be worth extending their description in this paragraph why motor learning (considered to take place at climbing fiber-Purkinje cell dendrites) is affected more obviously than the fear conditioning (considered to be mediated by mossy fiber ending, as described). Authors may be able to discuss this in a line that the former is more affected than the latter in SCA6 or other diseases they studied.

4. While paper is thorough in listing different comparisons, overall clarity of manuscript could be improved. For instance, adding more information to figures and figure legends to help each of them stand on their own. Specifically, the figures are inconsistent with respect to illustrating comparisons and reporting significance, with some having p values and brackets and others not. Similarly, some show individual data points with the expected value and variance (best) and others are just bar graphs better suited to a table. Please ensure that data presentation is uniform.

5. In figure 4 please add SCR during habituation or rest data, similar to how in figure 3 there is a "prior acquisition" part

6. In figures 6-10 authors use "no surviving voxels" but they do not use the same term in the results. Could authors please define in results what they mean by "no surviving voxels"? It is also confusing that there are no surviving voxels when in Fig 6E comparing the responses in A and C which both have different responses?

7. Figures 6 - 10 could be replaced by a table with major conclusions and these figures could be in supplemental. This is a suggestion.

Minor comments:

1. For Fig 1 please include how long did each block last.

2. In Figure 11, change the pre-, early-, and late-onset to the age time points.

3. Please define meaning of "Cue Retrieval" in the legend of Figure 11.

4. In methods please add that the anxiety questionnaire was adapted and include information on whether scores were adapted for tremor both in SCA patients and controls.

5. Please check "Figure 10" in Page 15, line 378, is correct.

6. Lines 669-672: no data is presented to show that there are no differences in the early fear acquisition training.

7. Line 126: References figure 11 for the experimental paradigm. This should be corrected to reference figure 1 which is the correct paradigm for human participants.

8. Line 192-193: A and B panels are not clearly labeled in the figure this line references.

9. Page 41, the first paragraph: "this differs from findings in motor associative learning, such as eyeblink conditioning" I recommend adding locomotor adaptation (for example, split-belt walking: Cerebellum (2018) 17:111-121) and hand reaching task (for example, prism adaption: Proc Natl Acad Sci U S A. 2018 Jul 10;115(28):7428-7433.) to eyeblink.

## Author Response

Synthesis of Reviews:

Synthesis Statement for Author (Required): Major comments:

1. Do the mice they used indeed show degeneration? If yes, please describe briefly, not just by citing previous paper, the degree of cerebellar cortical degeneration. If not, their descriptions in Abstract and elsewhere should be carefully modified. For example, in Page 4 line 80, we recommend "performed using a transgenic mouse model of SCA6 which presents cerebellar cortical degenerations in humans." Authors' response:

Yes, the SCA6 mouse model is a model of cerebellar cortical degeneration. This SCA6 mouse model is characterized by the expression of the P/Q-type calcium channel carboxy-terminus (CT) containing 27 polyglutamine repeats in specifically Purkinje cells, the same number of polyglutamine repeats found in the human disease for SCA6, which is referred to as CT-longQ27PC. Overexpression of the long CT-fragment in Purkinje cells resulted in Purkinje cell loss starting at 4 months of age which augmented with age and ataxia starting at 8 months of age. In contrast, control mice expressing the short CT fragments in Purkinje cells did not exhibit any Purkinje cell degeneration or ataxia symptoms up until the age of 18 months (Mark et al. 2015). Moreover, CT-longQ27PC mice displayed an increase in Purkinje cell aggregates in lobules 2, 6 and 8 during late stages (13-16 months of age) of the SCA6 disease compared to control mice expressing the short CT fragments (Bohne et al. 2022). As suggested, a brief description has been added to the revised introduction.

References:

Bohne P, Rybarski M, Mourabit DB-E, Krause F, Mark MD (2022) Cerebellar contribution to threat probability in a SCA6 mouse model. Hum Mol Gen 31:3807-3828.

Mark MD, Krause M, Boele HJ, Kruse W, Pollok S, Kuner T, Dalkara D, Koekkoek S, De Zeeuw CI, Herlitze S (2015) Spinocerebellar ataxia type 6 protein aggregates cause deficits in motor learning and cerebellar plasticity. J Neurosci 35:8882-8895. 2. Authors combine data from five different cerebellar ataxias and different stages of disease. Yet different SCAs may have dysfunction/pathology in different regions of the cerebellum, and later stages of disease also have worse pathology. Could authors please analyze the FC and MRI data per different stage of disease (e.g. per SARA score or disease duration) and for each type of ataxia? Further, we wondered if the manuscript could assay the degree of ataxia severity relative to the fear responses in SCA6 subjects (n=6, Table 1). Finally, the manuscript would be stronger for detailing any points consistent with SCA6 patients and transgenic mice? Authors' response:

These points are well taken. There are, however, constraints which are inherent to human cerebellar lesion studies. Patients with cerebellar disease are rare, and the patient pool in this study was even more limited given the exclusion criteria of the 7T MRI study. Therefore, different to studies in mice, we were unable to perform the study in patients with the same disease entity and comparing different disease stages. Please note, however, that we carefully chose disease entities. Only diseases which lead to pure cerebellar cortical degeneration, i.e., leaving other brain parts mostly intact, were included. Additional VBM analysis in the three major subgroups was performed and showed that patients with SCA6 (n=6), ADCAIII (n=5) and SAOA (n=6) showed very similar patterns of cerebellar degeneration, i.e., vermal areas and the anterior parts of the cerebellum were most severely degenerated. VBM analysis of subgroups has been included in Figure 6-1.

Furthermore, most patients (n=12) had a moderate degree of cerebellar ataxia based on the SARA score (SARA score > 10 and < 20; Lai et al. 2019; Yang et al. 2020). Only 5 patients had mild (SARA <= 10) and only 3 patients had severe ataxia (SARA >= 20). Sample sizes were too small to allow for reliable comparisons between disease stages. We redid, however, Figures 3,4 and 5 and color-coded individual patients based on SARA score (See Figures 3-1, 4-2, 5-3 and 5-4).

Skin conductance responses (SCRs) are variable already in controls. Analysis in small subgroups of participants is not meaningful. We followed, however, the advice of the reviewers, and plotted the behavioral data in the six SCA6 patients. Numerically, the findings were very similar to the group of all patients. Most importantly, i) SCA6 patients learned the dissociation between CS+ and CS- during acquisition training; based on questionnaires, responses to the CS+ were not significantly different to the CS- at the end of extinction training; and iii) differences in SCR incidences to the CS+ and CS- remained till the end of the recall phase. In the revised Figures 3,4 and 5 (see response to #6) individual data of the SCA6 patients are indicated in yellow.

We added to limitations that direct comparisons between the human and the mouse study is limited, because the human study was not restricted to patients with SCA6 and did not allow us to compare different disease stages.. References:

Lai RY, Tomishon D, Figueroa KP, Pulst SM, Perlman S, Wilmot G, Gomez CM, Schmahmann JD, Paulson H, Shakkottai VG, Ying SH, Zesiewicz T, Bushara K, Geschwind M, Xia G, Subramony SH, Ashizawa T, Kuo SH (2019) Tremor in the Degenerative Cerebellum: Towards the Understanding of Brain Circuitry for Tremor. Cerebellum 18:519-526.

Yang CY et al. (2020) Dysphagia in spinocerebellar ataxias type 1, 2, 3 and 6. J Neurol Sci 415:116878. 3. It is intriguing to know that fear-conditioning was not dramatically affected compared to these motor learning capabilities in ataxia patients. If the authors view is correct, it may be worth extending their description in this paragraph why motor learning (considered to take place at climbing fiber-Purkinje cell dendrites) is affected more obviously than the fear conditioning (considered to be mediated by mossy fiber ending, as described). Authors may be able to discuss this in a line that the former is more affected than the latter in SCA6 or other diseases they studied. Authors' response:

We followed the suggestion of the Reviewers and added the following paragraph to the revised discussion: "Furthermore, the cerebellar circuitry underlying fear conditioning and eyeblink conditioning may be distinct, and differentially affected in cerebellar cortical degeneration. For example, whereas long term depression (LTD) at the parallel fiber-Purkinje cell synapse is known to contribute to eyeblink conditioning (Boele et al. 2018), long-term potentiation (LTP) has been shown to be involved in fear conditioning (Sacchetti et al. 2005), with climbing fiber input being involved in LTD but not LTP." References:

Sacchetti B, Scelfo B, Strata P (2005) The cerebellum: synaptic changes and fear conditioning. Neuroscientist 11:217-227.

Boele HJ, Peter S, Ten Brinke MM, Verdonschot L, ACH IJ, Rizopoulos D, Gao Z, Koekkoek SKE, De Zeeuw CI (2018) Impact of parallel fiber to Purkinje cell long-term depression is unmasked in absence of inhibitory input. Sci Adv 4:eaas9426. 4. While paper is thorough in listing different comparisons, overall clarity of manuscript could be improved. For instance, adding more information to figures and figure legends to help each of them stand on their own. Specifically, the figures are inconsistent with respect to illustrating comparisons and reporting significance, with some having p values and brackets and others not. Similarly, some show individual data points with the expected value and variance (best) and others are just bar graphs better suited to a table. Please ensure that data presentation is uniform. Authors' response:

Thank you for the helpful remark. Figures have been edited to increase consistency. Individual data points have been added to the graphs. Figure legends were edited as suggested by the Reviewers. 5. In figure 4 please add SCR during habituation or rest data, similar to how in figure 3 there is a "prior acquisition" part.

Authors' response:

Done. Respective data has been added to the revised figures. 6. In figures 6-10 authors use "no surviving voxels" but they do not use the same term in the results. Could authors please define in results what they mean by "no surviving voxels"? It is also confusing that there are no surviving voxels when in Fig 6E comparing the responses in A and C which both have different responses? Authors' response:

Thank you for the helpful remark. We agree, "no surviving voxels" was not defined. In the revised manuscript we use the term "no surviving clusters" throughout, which refers to absence of clusters of equal or more than 10 voxels (isotropic voxel size: 1.7 mm) after application of threshold-free cluster-enhancement (TFCE) at p < 0.05 family-wise error (FWE) corrected level.

We agree with the Reviewers that the absence of significant group differences between patients and controls in Figure 6E (now Figure 7E) is confusing. However, it is important to note that we show TFCE corrected data, with TFCE correction enhancing clusters which based on individual voxels may not reach the significance threshold. TFCE correction takes values of neighboring voxels into account which is different to voxel-based correction. Despite the widespread activations in the control group, activations remain relatively weak. Given that data in the middle-aged participants and in the patients also show some degree of noise, the differential contrast likely shows no significant group difference, despite clear differences based on the figures. This explanation has been added to the revised figure legend. 7. Figures 6 - 10 could be replaced by a table with major conclusions and these figures could be in supplemental. This is a suggestion. Authors' response:

The authors believe that it is important to see the localization of cerebellar activations on a cerebellar flatmap. This is very difficult to represent in a table. No changes were made. Minor comments:

1. For Fig 1 please include how long did each block last.

Authors' response:

Done. In the revised Figure 1, we have added the duration of each block. 2. In Figure 11, change the pre-, early-, and late-onset to the age time points. Authors' response:

We have changed the age group terminology as suggested in figure (Figure 11, now Figure 12) legends, manuscript text and tables. We maintained the reference to disease stage across all age groups to facilitate comprehension and correlation of results to the human study. Additionally, we replaced "early onset" and "late onset" with "early stage" and "late stage" respectively, to align with human terminology. We have added the age of all mouse groups in parentheses under the disease stages in the figure as requested. 3. Please define meaning of "Cue Retrieval" in the legend of Figure 11. Authors' response:

In Figure 11 (now Figure 12) legend we added, "Freezing behavior during cue retrieval, which is defined as the percentage of freezing to the first two CS presentations during early extinction, was analyzed to show fear related learning to the cue and..." 4. In methods please add that the anxiety questionnaire was adapted and include information on whether scores were adapted for tremor both in SCA patients and controls. Authors' response:

We believe that this aspect was not adequately explained in the manuscript. We didn't change the questionnaire itself. However, we hypothesized that a question in the DASS-21 anxiety subscale, which asked about whether the participants experienced trembling, might have been affecting patients' anxiety scores. This could explain the close-to-significant differences (p = 0.064, Mann-Whitney-U test) between groups. So, we decided to reanalyze data in the questionnaire by excluding answers to this particular question (in both patients and controls). As we expected, these close-to-significant differences disappeared (p = 0.153, Mann-Whitney-U test). We provided clarification for this in the figure legend. 5. Please check "Figure 10" in Page 15, line 378, is correct.

Authors' response:

Done. The mistake has been corrected. 6. Lines 669-672: no data is presented to show that there are no differences in the early fear acquisition training. Authors' response:

The absence of differences in early acquisition training is presented in Table 6-2. We included a reference to the table in the revised version of the manuscript. 7. Line 126: References figure 11 for the experimental paradigm. This should be corrected to reference figure 1 which is the correct paradigm for human participants.

Authors' response:

Done. Thank you for pointing this out. 8. Line 192-193: A and B panels are not clearly labeled in the figure this line references.

Authors' response:

Done. We added explicit information about the context used (that is, acquisition context, extinction context). 9. Page 41, the first paragraph: "this differs from findings in motor associative learning, such as eyeblink conditioning" I recommend adding locomotor adaptation (for example, split-belt walking: Cerebellum (2018) 17:111-121) and hand reaching task (for example, prism adaption: Proc Natl Acad Sci U S A. 2018 Jul 10;115(28):7428-7433.) to eyeblink.

Authors' response:

Done. The discussion has been revised accordingly.
