# Supplementary figures and images for: Mild Deficits in Fear Learning: Evidence from Humans and Mice with Cerebellar Cortical Degeneration
Source: eNeuro. 2024 Feb 22;11(2):ENEURO.0365-23.2023. doi: 10.1523/ENEURO.0365-23.2023 (PMC10897646; doi:10.1523/ENEURO.0365-23.2023)

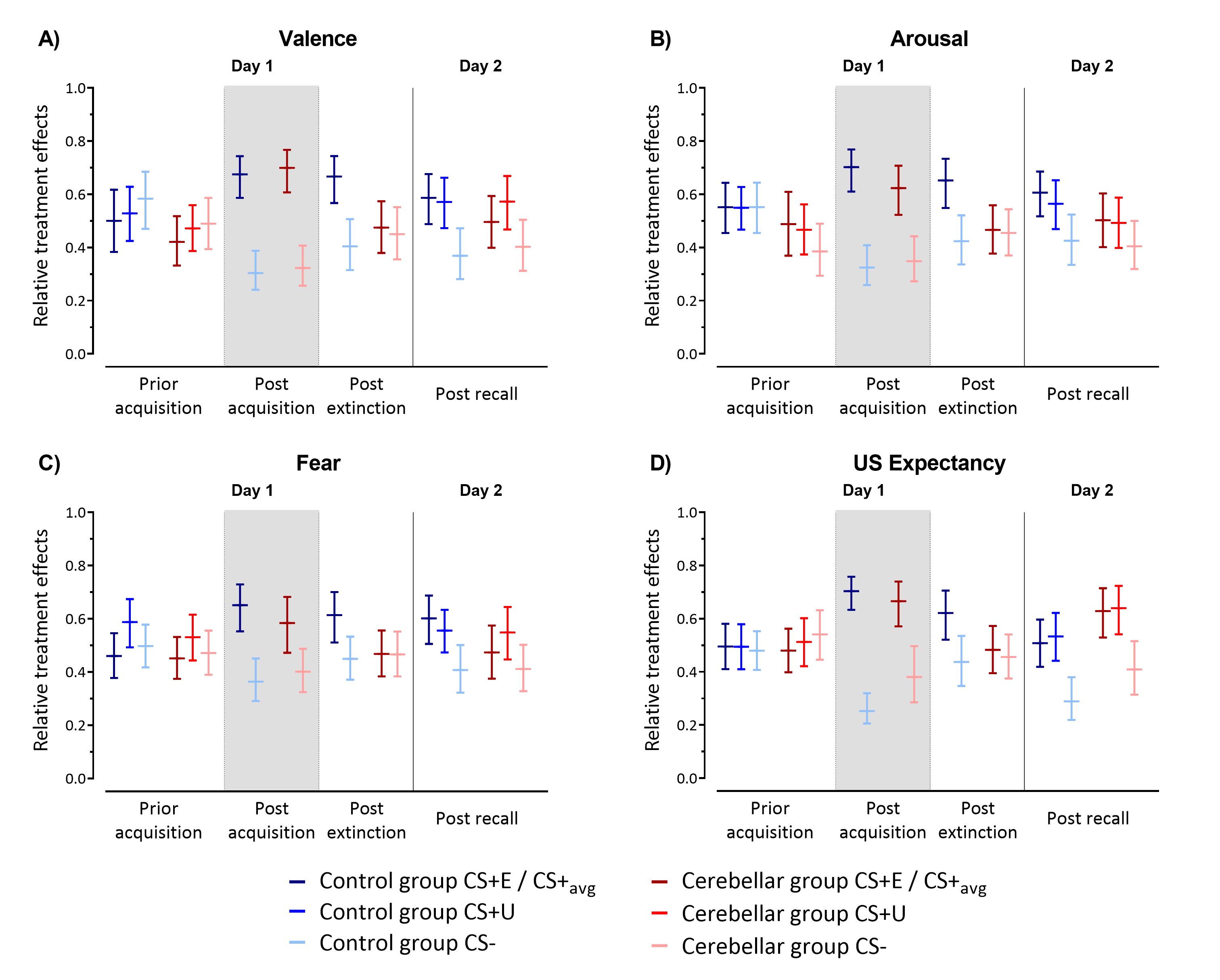

Supplement: Figure 4-1 — Relative treatment effect (RTE) estimates for A) valence, B) arousal, C) fear and D) US expectancy. Horizontal lines denote median RTEs and whiskers denote 95% confidence intervals. Blue colors = controls, red colors = cerebellar patients. Dark colors: CS+E and CS+U, light colors: CS–. Gray background = fear acquisition training. Post acquisition training responses to CS+E and CS+U were averaged (CS+avg). Download Figure 4-1, TIF file. [file eneuro-11-ENEURO.0365-23.2023-s011.tif]

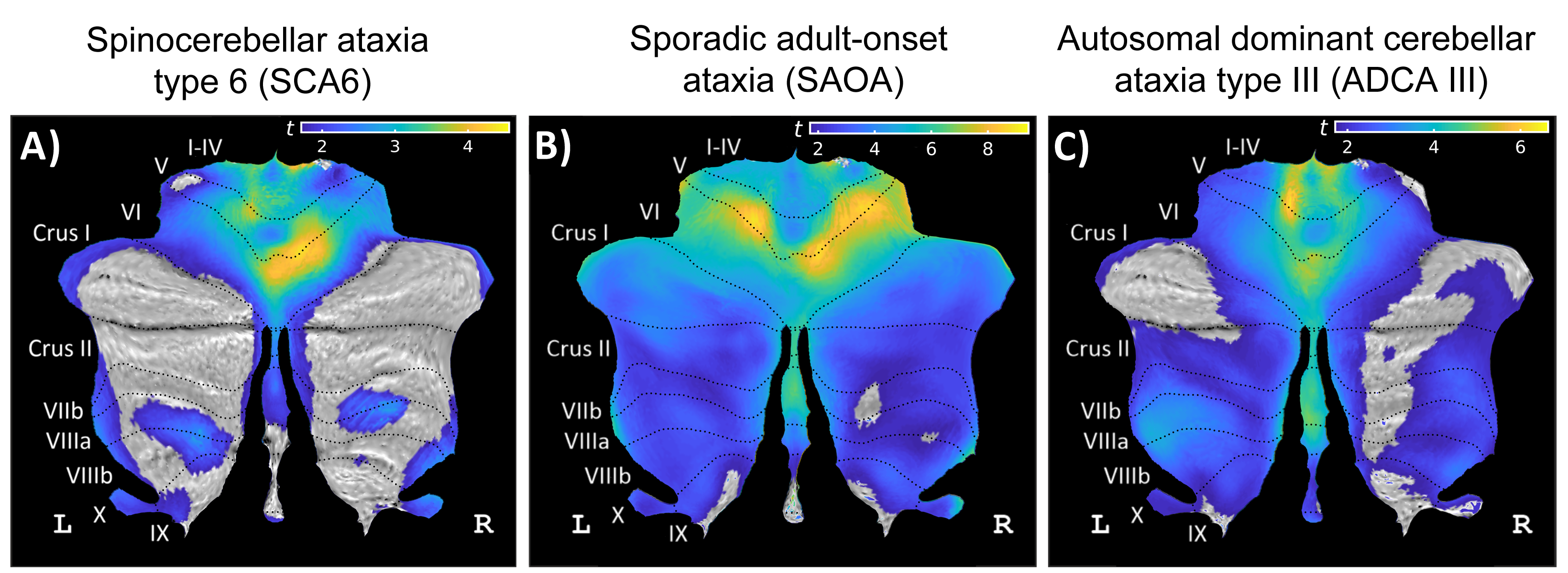

Supplement: Figure 6-1 — Gray matter voxel-based morphometry [contrast ‘control group > patient group’] for patient subgroups with A) spinocerebellar ataxia type 6 (SCA6, n = 6), B) sporadic adult-onset ataxia of unknown etiology (SAOA, n = 6) and C) autosomal dominant cerebellar ataxia type III (ADCA III, n = 5, SCA6 patients are not included in the ADCA III group). VBM group results are calculated using an uncorrected threshold p < 0.05 and in MNI space projected on a cerebellar flatmap using the SUIT toolbox (Diedrichsen and Zotow, 2015). VBM = voxel-based morphometry; L = left; R = right; SUIT = spatially unbiased atlas template of the cerebellum. Download Figure 6-1, TIF file. [file eneuro-11-ENEURO.0365-23.2023-s017.tif]
