# Supplementary material for: Mild Deficits in Fear Learning: Evidence from Humans and Mice with Cerebellar Cortical Degeneration
Source: eNeuro. 2024 Feb 22;11(2):ENEURO.0365-23.2023. doi: 10.1523/ENEURO.0365-23.2023 (PMC10897646; doi:10.1523/ENEURO.0365-23.2023)
Supplement: Table 4-1 — Results of the non-parametric ANOVA-type statistics for repeated measures for valence, arousal, fear and US expectancy ratings comparing cerebellar patient and control groups. Download Table 4-1, DOC file. [file eneuro-11-ENEURO.0365-23.2023-s002.doc]

## Table 4-1. Results of the non-parametric ANOVA-type statistics for repeated measures for valence, arousal, fear and US expectancy ratings comparing cerebellar patient and control groups.

| **Factor** | **Numerator Df** | ***F*** | ***P*** |
| --- | --- | --- | --- |
| **Valence** | | | |
| Group  Stimulus  Time  Group × Stimulus  Group × Time  Stimulus × Time  Group × Stimulus × Time | 1  1.66  2.84  1.66  2.84  4.1  4.1 | 0.45  17.60  13.71  0.90  0.68  20.03  1.68 | 0.504  **<.001*****  **<.001*****  0.390  0.558  **<.001*****  0.149 |
| **Arousal** | | | |
| Group  Stimulus  Time  Group × Stimulus  Group × Time  Stimulus × Time  Group × Stimulus × Time | 1  1.81  2.69  1.81  2.69  3.83  3.83 | 1.66  18.98  9.77  0.83  0.24  6.55  2.35 | 0.198  **<.001*****  **<.001*****  0.425  0.850  **<.001*****  0.054 |
| **Fear** | | | |
| Group  Stimulus  Time  Group × Stimulus  Group × Time  Stimulus × Time  Group × Stimulus × Time | 1  1.66  2.59  1.66  2.59  4.29  4.29 | 0.59  16.41  10.38  2.60  0.10  4.88  1.07 | 0.444  **<.001*****  **<.001*****  0.085  0.943  **<.001*****  0.373 |
| **US expectancy** | | | |
| Group  Stimulus  Time  Group × Stimulus  Group × Time  Stimulus × Time  Group × Stimulus × Time | 1  1.73  2.51  1.73  2.51  4.21  4.21 | 0.39  31.14  23.55  2.13  1.76  14.01  0.70 | 0.531  **<.001*****  **<.001*****  0.126  0.163  **<.001*****  0.597 |

*** Significant results at *p* < 0.001.
