# Supplementary material for: Mild Deficits in Fear Learning: Evidence from Humans and Mice with Cerebellar Cortical Degeneration
Source: eNeuro. 2024 Feb 22;11(2):ENEURO.0365-23.2023. doi: 10.1523/ENEURO.0365-23.2023 (PMC10897646; doi:10.1523/ENEURO.0365-23.2023)
Supplement: Table 5-1 — Results of the non-parametric ANOVA-type statistics for repeated measures for skin conductance response (SCR) amplitudes comparing cerebellar patient and control groups. Download Table 5-1, DOC file. [file eneuro-11-ENEURO.0365-23.2023-s003.doc]

**Table 5-1. Results of the non-parametric ANOVA-type statistics for repeated measures for skin conductance response (SCR) amplitudes comparing cerebellar patient and control groups.**

| **Factor** | **Numerator Df** | ***F*** | ***P*** |
| --- | --- | --- | --- |
| **CS-related skin conductance responses** | | | |
| **CS+_avg_ (average CS+E and CS+U) vs CS-** | | | |
| *Fear acquisition training* | | | |
| Stimulus  Block  Group  Stimulus × Block  Block × Group  Stimulus × Group  Stimulus × Block × Group | 1  1  1  1  1  1  1 | 14.13  23.77  0.04  0.01  0.05  1.74  0.95 | **<.001*****  **<.001*****  0.850  0.936  0.828  0.187  0.330 |
| **CS+E vs CS+U vs CS-** | | | |
| *Habituation* | | | |
| Stimulus  Group  Stimulus × Group | 1.66  1  1.66 | 2.27  0.22  0.08 | 0.114  0.640  0.896 |
| *Fear acquisition training* | | | |
| Stimulus  Block  Group  Stimulus × Block  Block × Group  Stimulus × Group  Stimulus × Block × Group | 1.97  1  1  1.91  1  1.97  1.91 | 2.24  25.91  <0.01  0.04  0.04  3.09  0.53 | 0.108  **<.001*****  0.961  0.953  0.838  **0.046***  0.581 |
| *Extinction training* | | | |
| Stimulus  Block  Group  Stimulus × Block  Block × Group  Stimulus × Group  Stimulus × Block × Group | 1  1  1  1  1  1  1 | 1.04  11.03  0.03  1.99  0.18  0.01  0.01 | 0.308  **<.001*****  0.857  0.158  0.675  0.904  0.906 |
| *Recall* | | | |
| Stimulus  Block  Group  Stimulus × Block  Block × Group  Stimulus × Group  Stimulus × Block × Group | 1.92  1  1  1.87  1  1.92  1.87 | 4.68  11.51  0.09  3.74  0.02  2.25  0.83 | **0.010***  **<.001*****  0.770  **0.027***  0.892  0.108  0.427 |
| **US-related skin conductance responses (paired CS+_avg_ vs unpaired CS+_avg_ vs CS-)** | | | |
| *Fear acquisition training* | | | |
| Stimulus  Group  Stimulus×Group | 1.87  1  1.87 | 106.04  0.02  0.73 | **<.001*****  0.886  0.475 |

* Significant results at *p* < 0.05.

*** Significant results at *p* < 0.001.

*Post-hoc* analysis of significant Stimulus × Group interaction during acquisition training performed on the data with separate CS+E vs CS+U vs CS- stimuli revealed significantly higher SCR amplitudes towards CS+E comparing to CS- trials in cerebellar group, however, differences did not survive correction for multiple comparisons (*p* = 0.067, least squares means test).
