# Supplementary material for: Mild Deficits in Fear Learning: Evidence from Humans and Mice with Cerebellar Cortical Degeneration
Source: eNeuro. 2024 Feb 22;11(2):ENEURO.0365-23.2023. doi: 10.1523/ENEURO.0365-23.2023 (PMC10897646; doi:10.1523/ENEURO.0365-23.2023)
Supplement: Table 5-2 — Results of the non-parametric ANOVA-type statistics for repeated measures for skin conductance response (SCR) incidences between patient and control groups. Download Table 5-2, DOC file. [file eneuro-11-ENEURO.0365-23.2023-s004.doc]

## Table 5-2. Results of the non-parametric ANOVA-type statistics for repeated measures for skin conductance response (SCR) incidences between patient and control groups.

| **Factor** | **Numerator Df** | ***F*** | ***P*** |
| --- | --- | --- | --- |
| **CS+_avg_ (average CS+E and CS+U) vs CS-** | | | |
| *Fear acquisition training* | | | |
| Stimulus  Block  Group  Stimulus × Block  Block × Group  Stimulus × Group  Stimulus × Block × Group | 1  1  1  1  1  1  1 | 46.99  24.66  0.39  0.07  0.15  0.04  8.28 | **<.001*****  **<.001*****  0.534  0.794  0.697  0.835  **0.004*** |
| **CS+E vs CS+U vs CS-** | | | |
| *Habituation* | | | |
| Stimulus  Group  Stimulus × Group | 1.95  1  1.95 | 0.68  0.08  0.19 | 0.505  0.779  0.818 |
| *Fear acquisition training* | | | |
| Stimulus  Block  Group  Stimulus × Block  Block × Group  Stimulus × Group  Stimulus × Block × Group | 1.96  1  1  1.89  1  1.96  1.89 | 0.83  27.79  0.29  0.11  0.23  3.21  4.39 | 0.435  **<.001*****  0.587  0.883  0.630  **0.042***  **0.014*** |
| *Extinction training* | | | |
| Stimulus  Block  Group  Stimulus × Block  Block × Group  Stimulus × Group  Stimulus × Block × Group | 1  1  1  1  1  1  1 | 1.14  12.33  <0.01  2.81  0.13  0.72  0.43 | 0.286  **<.001*****  0.998  0.094  0.717  0.396  0.514 |
| *Recall* | | | |
| Stimulus  Block  Group  Stimulus × Block  Block × Group  Stimulus × Group  Stimulus × Block × Group | 1.9  1  1  1.88  1  1.9  1.88 | 4.03  7.29  0.66  2.89  0.04  3.33  0.52 | **0.019***  **0.007***  0.418  0.059  0.848  **0.038***  0.585 |

* Significant results at *p* < 0.05.

*** Significant results at *p* < 0.001.

*Post-hoc* analysis of significant Stimulus × Group interaction during acquisition training performed on the data with separate CS+E vs CS+U vs CS- stimuli did not reveal any significant differences (all *p* values ≥ 0.442, least squares means test). *Post-hoc* analysis of Stimulus × Block × Group interaction during acquisition training performed on the data with separate CS+E vs CS+U vs CS- stimuli revealed significantly lower SCR incidences in late compared to the early CS- block in patients (*p* = 0.003, least squares means test), significantly higher SCR incidences in late CS+E compared to the late CS- block in patients (*p* = 0.002, least squares means test) and significantly higher SCR incidences in late CS+E and CS+U blocks compared to the respective early CS+E and CS+U blocks in controls (both *p* values ≤ 0.021, least squares means test).
