# Supplementary material for: Mild Deficits in Fear Learning: Evidence from Humans and Mice with Cerebellar Cortical Degeneration
Source: eNeuro. 2024 Feb 22;11(2):ENEURO.0365-23.2023. doi: 10.1523/ENEURO.0365-23.2023 (PMC10897646; doi:10.1523/ENEURO.0365-23.2023)
Supplement: Table 6-1 — Voxel-based morphometry results. Gray matter clusters are reported which were significant after application of threshold-free cluster-enhancement (TFCE) at p < 0.05 FWE corrected level (second level t-test). One cluster is detected and displayed (isotropic voxel size: 0.6 mm). For this cluster, three local maxima are listed separated by at least 8 mm. Download Table 6-1, DOC file. [file eneuro-11-ENEURO.0365-23.2023-s005.doc]

Table 6-1. Voxel-based morphometry results. Gray matter clusters are reported which were significant after application of threshold-free cluster-enhancement (TFCE) at *p* < 0.05 FWE corrected level (second level *t*-test). One cluster is detected and displayed (isotropic voxel size: 0.6 mm). For this cluster, three local maxima are listed separated by at least 8 mm.

| Index | Location (lobule) | Side | MNI coordinates / mm | | | Cluster size/mm^3^ | *p_FWE_* | TFCE |
| --- | --- | --- | --- | --- | --- | --- | --- | --- |
| *healthy controls > patients* | | | | | | | | |
| 1 | Right_V  Right_VI  Right_I_IV | right  right  right | 6  10  8 | -64.5  -64.5  -52 | -21  -14  -20.5 | 180685.125 | < 0.001  < 0.001  < 0.001 | 74689.08  74066.06  72826.1 |
| *healthy controls < patients* | | | | | | | | |
|  | no surviving clusters | | | | | | | |
