# Supplementary material for: Mild Deficits in Fear Learning: Evidence from Humans and Mice with Cerebellar Cortical Degeneration
Source: eNeuro. 2024 Feb 22;11(2):ENEURO.0365-23.2023. doi: 10.1523/ENEURO.0365-23.2023 (PMC10897646; doi:10.1523/ENEURO.0365-23.2023)
Supplement: Table 6-2 — Fear acquisition training, extinction training and recall. Activation clusters are reported which were significant after application of threshold-free cluster-enhancement (TFCE) at p < 0.05 FWE corrected level (t-tests). Displayed are all clusters ≥ 10 voxel (isotropic voxel size: 1.7 mm). In each cluster, up to three maxima are listed separated by at least 8 mm. CS+ = CS+E and CS+U trials were collapsed into the CS+. ncl. = nucleus, pFWE = family-wise error p value. Download Table 6-2, DOC file. [file eneuro-11-ENEURO.0365-23.2023-s006.doc]

Table 6-2. Fear acquisition training, extinction training and recall. Activation clusters are reported which were significant after application of threshold-free cluster-enhancement (TFCE) at *p* < 0.05 FWE corrected level (*t*-tests). Displayed are all clusters ≥ 10 voxel (isotropic voxel size: 1.7 mm). In each cluster, up to three maxima are listed separated by at least 8 mm. CS+ = CS+E and CS+U trials were collapsed into the CS+. ncl. = nucleus, *p_FWE_* = family-wise error *p* value.

| Index | Location / lobule  (**Cluster size / voxel**) | Side | SUIT coordinates / mm | | | *p_FWE_* | TFCE |
| --- | --- | --- | --- | --- | --- | --- | --- |
| *healthy controls, acquisition training, US post CS+ > no-US post CS-* | | | | | | | |
| 1 | Extended cluster (**23869**) | left VI (2298), right VI (2273), white matter (1767), right Crus I (1613), left Crus I (1440), left Crus II (1211), left V (1084), right V (1019), left VIIIa (990), right I-IV (975), left I-IV (955), left VIIb (916), right Crus II (913), right VIIIa (890), left VIIIb (864), right VIIIb (852), right VIIb (702), right IX (534), vermal VI (529), left IX (404), vermal VIIIa (319), left dentate ncl. (249), right dentate ncl. (240), vermal IX (197), vermal VIIIb (159), right X (107), vermal Crus II (106), left X (95), vermal VIIb (48), right interposed ncl. (37), left interposed ncl. (35), vermal X (24), left fastigial ncl. (10), right fastigial ncl. (9), vermal Crus I (5) | | | | | |
|  | VIIIa | vermal | 3.2 | -62.7 | -32.6 | < 0.001 | 4490 |
|  | VIIIb | vermal | 3.2 | -64.4 | -47.9 | < 0.001 | 4311 |
|  | VI | left | -25.7 | -64.4 | -19.0 | < 0.001 | 4095 |
| *patients, acquisition training, US post CS+ > no-US post CS-* | | | | | | | |
| 1 | Extended cluster (**16809**) | white matter (1639), right VI (1500), left VI (1302), left VIIIa (1053), left V (1034), right V (1008), left I-IV (890), right VIIIb (884), left VIIIb (876), right I-IV (830), right Crus II (830), right VIIIa (817), left VIIb (660), right VIIb (566), right IX (417), vermal VIIIa (319), vermal VI (311), right dentate ncl. (246), right Crus I (243), left Crus II (206), vermal IX (183), left dentate ncl. (182), left Crus I (168), vermal VIIIb (159), left IX (151), vermal Crus II (68), right interposed ncl. (58), vermal VIIb (48), left interposed ncl. (48), right X (45), left X (32), vermal X (20), left fastigial ncl. (8), right fastigial ncl. (8) | | | | | |
|  | I-IV | left | -13.8 | -42.3 | -19.0 | < 0.001 | 3349 |
|  | VIIIa | vermal | 4.9 | -62.7 | -30.9 | < 0.001 | 3256 |
|  | I-IV | left | -8.7 | -40.6 | -25.8 | < 0.001 | 3170 |
| *healthy controls > patients, acquisition training,* no-US post CS+ > no US post CS- | | | | | | | |
|  | no surviving clusters | | | | | | |
| *healthy controls, acquisition training,* no-US post CS+ *> rest* | | | | | | | |
| 1 | Extended cluster (**289**) | left Crus I (172), left VI (83), left Crus II (34) | | | | | |
|  | Crus I | left | -12.1 | -78.0 | -27.5 | 0.01 | 776 |
|  | Crus I | left | -27.4 | -67.8 | -30.9 | 0.018 | 689 |
| 2 | Crus I (**19**) | left | -37.6 | -62.7 | -32.6 | 0.043 | 541 |
| *patients, acquisition training,* no-US post CS+ *> rest* | | | | | | | |
| 1 | Extended cluster (**2998**) | left Crus I (865), left VI (676), left VIIb (645), left Crus II (535), white matter (130), left VIIIa (90), vermal Crus II (25), vermal VI (24), vermal Crus I (5), left dentate ncl. (3) | | | | | |
|  | VIIb | left | -37.6 | -61.0 | -51.3 | < 0.001 | 1189 |
|  | VIIb | left | -37.6 | -66.1 | -58.1 | < 0.001 | 1142 |
|  | VIIb | left | -29.1 | -71.2 | -56.4 | 0.001 | 1071 |
| 2 | Extended cluster (**292**) | left IX (142), left VIIIb (89), left X (41), left VIIIa (11), white matter (9) | | | | | |
|  | IX | left | -7.0 | -52.5 | -51.3 | 0.019 | 568 |
|  | VIIIb | left | -22.3 | -38.9 | -47.9 | 0.027 | 519 |
|  | IX | left | -12.1 | -45.7 | -51.3 | 0.039 | 465 |
| 3 | Crus I (**49**) | right | 44.0 | -61.0 | -29.2 | 0.029 | 509 |
| *healthy controls > patients, acquisition training,* no-US post CS+ *> rest* | | | | | | | |
|  | no surviving clusters | | | | | | |
| *patients > healthy controls, acquisition training,* no-US post CS+ *> rest* | | | | | | | |
|  | no surviving clusters | | | | | | |
| *healthy controls, early acquisition training, CS+ > rest* | | | | | | | |
|  | no surviving clusters | | | | | | |
| *healthy controls, early acquisition training, CS- > rest* | | | | | | | |
|  | no surviving clusters | | | | | | |
| *healthy controls, late acquisition training, CS+ > rest* | | | | | | | |
| 1 | Extended cluster (**2653**) | left VI (774), left Crus I (465), right VI (314), vermal VI (208), white matter (174), left Crus II (151), vermal IX (96), left IX (62), left dentate ncl. (60), vermal VIIIa (59), vermal Crus II (41), right Crus I (38), right V (32), left V (29), right interposed ncl. (29), right IX (23), vermal VIIb (16), right dentate ncl. (16), vermal VIIIb (13), right Crus II (11), left interposed ncl. (11), left VIIb (6), right VIIb (6), left VIIIb (6), vermal Crus I (5), right fastigial ncl. (4), right VIIIb (2), right I-IV (1), left VIIIa (1) | | | | | |
|  | Crus II | left | -13.8 | -74.6 | -36.0 | 0.003 | 838 |
|  | VI | left | -8.7 | -69.5 | -29.2 | 0.003 | 837 |
|  | VI | left | -5.3 | -71.2 | -19.0 | 0.003 | 834 |
|  | Crus II | left | -13.8 | -74.6 | -36.0 | 0.003 | 838 |
| 2 | Extended cluster (**302**) | right Crus I (149), right VI (148), white matter (5) | | | | | |
|  | VI | right | 42.3 | -47.4 | -25.8 | 0.024 | 544 |
|  | VI | right | 37.2 | -55.9 | -22.4 | 0.031 | 506 |
|  | Crus I | right | 37.2 | -69.5 | -25.8 | 0.032 | 502 |
| 3 | I-IV (**10**) | left | -1.9 | -52.5 | 1.4 | 0.045 | 454 |
| *healthy controls, late acquisition training, CS- > rest* | | | | | | | |
| 1 | Extended cluster (**5391**) | left VI (1097), left Crus I (519), left VIIb (510), left Crus II (383), left V (364), vermal VI (253), left I-IV (251), right V (235), right VI (225), white matter (218), right Crus II (213), left VIIIb (212), right I-IV (198), left IX (197), right VIIb (98), left VIIIa (94), vermal IX (90), vermal Crus II (67), vermal VIIIa (63), vermal VIIb (34), vermal VIIIb (32), left interposed ncl. (16), right Crus I (7), left dentate ncl. (7), left X (6), vermal Crus I (2) | | | | | |
|  | VI | left | -34.2 | -50.8 | -22.4 | 0.001 | 1311 |
|  | V | left | -3.6 | -67.8 | -10.5 | 0.002 | 1112 |
|  | VI | left | -32.5 | -38.9 | -27.5 | 0.002 | 1108 |
| 2 | Extended cluster (**108**) | right VIIb (56), right VIIIa (52) | | | | | |
|  | VIIb | right | 35.5 | -61.0 | -53.0 | 0.04 | 548 |
|  | VIIb | right | 28.7 | -67.8 | -49.6 | 0.046 | 522 |
|  | VIIIa | right | 33.8 | -50.8 | -51.3 | 0.047 | 519 |
| 3 | VIIIb (**24**) | right | 16.8 | -47.4 | -49.6 | 0.043 | 536 |
| *patients, early acquisition training, CS+ > rest* | | | | | | | |
|  | no surviving clusters | | | | | | |
| *patients, early acquisition training, CS- > rest* | | | | | | | |
|  | no surviving clusters | | | | | | |
| *patients, late acquisition training, CS+ > rest* | | | | | | | |
| 1 | Extended cluster (**170**) | left VIIb (117), left Crus II (27), left VIIIa (22), white matter (2), left Crus I (2) | | | | | |
|  | VIIb | left | -35.9 | -57.6 | -51.3 | 0.022 | 631 |
|  | VIIb | left | -37.6 | -49.1 | -54.7 | 0.039 | 527 |
|  | Crus II | left | -32.5 | -59.3 | -42.8 | 0.047 | 497 |
| 2 | Extended cluster (**133**) | right VIIb (126), right VIIIa (7) | | | | | |
|  | VIIb | right | 23.6 | -74.6 | -54.7 | 0.022 | 627 |
|  | VIIb | right | 16.8 | -71.2 | -58.1 | 0.042 | 518 |
|  | VIIb | right | 37.2 | -67.8 | -58.1 | 0.047 | 497 |
| 3 | VIIb (**102**) | right | 35.5 | -54.2 | -49.6 | 0.024 | 612 |
| *patients, late acquisition training, CS- > rest* | | | | | | | |
|  | no surviving clusters | | | | | | |
| *healthy controls > patients, late acquisition training, CS+ > rest* | | | | | | | |
|  | no surviving clusters | | | | | | |
| *healthy controls > patients, late acquisition training, CS- > rest* | | | | | | | |
| 1 | Extended cluster (**106**) | left I-IV (63), right I-IV (36), left V (7) | | | | | |
|  | I-IV | right | 1.5 | -50.8 | -13.9 | 0.034 | 520 |
|  | V | left | -7.0 | -54.2 | -13.9 | 0.041 | 490 |
| *healthy controls, late > early acquisition training, CS+ > rest* | | | | | | | |
| 1 | Extended cluster (**5019**) | left Crus I (625), white matter (599), right VI (555), left VI (544), right Crus I (477), left V (437), right V (360), right I-IV (218), left I-IV (196), right IX (163), left Crus II (156), vermal VI (140), left IX (108), vermal VIIIa (74), right dentate ncl. (65), right X (52), left dentate ncl. (45), right VIIIb (31), vermal VIIb (26), right interposed ncl. (26), vermal IX (23), right VIIIa (22), right Crus II (19), left interposed ncl. (18), vermal Crus II (17), vermal X (8), right VIIb (6), vermal Crus I (3), left VIIb (2), vermal VIIIb (2), right fastigial ncl. (2) | | | | | |
|  | white matter |  | 28.7 | -49.1 | -39.4 | 0.013 | 422 |
|  | VIIIa | vermal | -1.9 | -62.7 | -30.9 | 0.014 | 413 |
|  | Crus I | left | -39.3 | -76.3 | -39.4 | 0.017 | 394 |
| *healthy controls, late > early acquisition training, CS-* | | | | | | | |
| 1 | Extended cluster (**5096**) | left VI (1280), left V (724), right V (715), right VI (540), left I-IV (366), right I-IV (285), white matter (267), vermal VI (248), right VIIb (84), left Crus I (76), right Crus II (70), vermal VIIIa (70), right VIIIa (64), vermal Crus II (63), right VIIIb (52), left Crus II (43), vermal IX (28), vermal VIIb (27), left interposed ncl. (25), right dentate ncl. (22), vermal VIIIb (13), left IX (13), left VIIb (8), left dentate ncl. (5), right IX (4), right interposed ncl. (2), right Crus I (1), right fastigial ncl. (1) | | | | | |
|  | V | left | -5.3 | -66.1 | -8.8 | 0.007 | 850 |
|  | V | right | 6.6 | -64.4 | -10.5 | 0.012 | 745 |
|  | I-IV | right | 4.9 | -55.9 | -8.8 | 0.013 | 723 |
| 2 | IX (**13**) | right | 15.1 | -50.8 | -51.3 | 0.046 | 455 |
| 3 | VIIIa (**32**) | left | -24.0 | -54.2 | -53.0 | 0.047 | 451 |
| 4 | VIIIa (**10**) | right | 28.7 | -54.2 | -51.3 | 0.048 | 444 |
| *patients, late > early acquisition training, CS+ > rest* | | | | | | | |
|  | no surviving clusters | | | | | | |
| *patients, late > early acquisition training, CS- > rest* | | | | | | | |
|  | no surviving clusters | | | | | | |
| *healthy controls, early extinction training No-US post CS+ > rest* | | | | | | | |
| 1 | Extended cluster (**1697**) | left Crus I (692), left VI (511), left Crus II (251), white matter (176), left VIIb (37), vermal VI (20), left VIIIa (5), vermal Crus I (4), vermal Crus II (1) | | | | | |
|  | VI | left | -27.4 | -66.1 | -30.9 | 0.009 | 655 |
|  | Crus I | left | -7.0 | -76.3 | -27.5 | 0.012 | 617 |
|  | Crus I | left | -32.5 | -57.6 | -32.6 | 0.012 | 617 |
| 2 | Extended cluster (**97**) | right IX (30), white matter (25), vermal IX (12), right interposed ncl. (12), vermal VIIIb (10), vermal VIIIa (6), right dentate ncl. (2) | | | | | |
|  | interposed ncl. | right | 6.6 | -57.6 | -32.6 | 0.029 | 486 |
|  | white matter |  | 13.4 | -55.9 | -41.1 | 0.046 | 416 |
| 3 | Extended cluster (**138**) | right VI (86), right Crus I (52) | | | | | |
|  | Crus I | right | 32.1 | -61.0 | -32.6 | 0.036 | 454 |
|  | VI | right | 25.3 | -67.8 | -29.2 | 0.036 | 453 |
|  | Crus I | right | 35.5 | -52.5 | -34.3 | 0.046 | 416 |
| 4 | IX (**47**) | right | 6.6 | -57.6 | -54.7 | 0.04 | 437 |
| 5 | Extended cluster (**75**) | right Crus I (46), right Crus II (21), white matter (8) | | | | | |
|  | Crus I | right | 18.5 | -76.3 | -30.9 | 0.04 | 436 |
|  | Crus II | right | 16.8 | -74.6 | -41.1 | 0.044 | 424 |
| 6 | VI (**66**) | right | 8.3 | -76.3 | -25.8 | 0.041 | 434 |
| *healthy controls, early extinction training No-US post CS- > rest* | | | | | | | |
|  | no surviving clusters | | | | | | |
| *patients, early extinction training No-US post CS+ > rest* | | | | | | | |
|  | no surviving clusters | | | | | | |
| *patients, early extinction training No-US post CS- > rest* | | | | | | | |
| 1 | Crus I (**78**) | left | -39.3 | -64.4 | -41.1 | 0.023 | 554 |
| 2 | Extended cluster (**113**) | left Crus I (63), left VI (50) | | | | | |
|  | VI | left | -34.2 | -61.0 | -27.5 | 0.034 | 498 |
|  | Crus I | left | -29.1 | -66.1 | -32.6 | 0.047 | 450 |
|  | VI | left | -27.4 | -55.9 | -30.9 | 0.048 | 446 |
| 3 | Extended cluster (**71**) | left VI (59), left Crus I (12) | | | | | |
|  | VI | left | -15.5 | -76.3 | -20.7 | 0.038 | 480 |
|  | Crus I | left | -22.3 | -74.6 | -25.8 | 0.043 | 463 |
| 4 | Extended cluster (**33**) | left Crus I (33) | | | | | |
|  | Crus I | left | -18.9 | -86.5 | -24.1 | 0.039 | 477 |
|  | Crus I | left | -12.1 | -81.4 | -24.1 | 0.048 | 446 |
| 5 | Crus I (**26**) | left | -49.5 | -69.5 | -29.2 | 0.04 | 475 |
| *patients, early extinction training No-US post CS- > rest* | | | | | | | |
|  | no surviving clusters | | | | | | |
| *healthy controls > patients, early extinction training No-US post CS+ > rest* | | | | | | | |
|  | no surviving clusters | | | | | | |
| *healthy controls > patients, early extinction training No-US post CS- > rest* | | | | | | | |
|  | no surviving clusters | | | | | | |
| *healthy controls, early recall CS+ > rest* | | | | | | | |
| 1 | VIIb (**275**) | left | -22.3 | -72.9 | -51.3 | 0.008 | 696 |
| 2 | Extended cluster (**177**) | left Crus II (87), left Crus I (60), left VI (30) | | | | | |
|  | Crus II | left | -3.6 | -79.7 | -34.3 | 0.028 | 506 |
|  | Crus II | left | -18.9 | -76.3 | -41.1 | 0.034 | 477 |
|  | VI | left | -7.0 | -74.6 | -27.5 | 0.035 | 476 |
| *healthy controls, early recall CS- > rest* | | | | | | | |
|  | no surviving clusters | | | | | | |
| *healthy controls, early recall No-US post CS+ > rest* | | | | | | | |
| 1 | VIIb (**41**) | left | -25.7 | -67.8 | -49.6 | 0.041 | 683 |
| 2 | Crus I (**25**) | left | -17.2 | -78.0 | -29.2 | 0.043 | 669 |
| *healthy controls, early recall No-US post CS- > rest* | | | | | | | |
|  | no surviving clusters | | | | | | |
| *patients, early recall CS+ > rest* | | | | | | | |
|  | no surviving clusters | | | | | | |
| *patients, early recall CS- > rest* | | | | | | | |
|  | no surviving clusters | | | | | | |
| *patients, early recall No-US post CS+ > rest* | | | | | | | |
|  | no surviving clusters | | | | | | |
| *patients, early recall No-US post CS- > rest* | | | | | | | |
|  | no surviving clusters | | | | | | |
| *healthy controls > patients, early recall, CS+ > rest* | | | | | | | |
| 1 | Extended cluster (**429**) | left Crus II (389), left Crus I (39), left VIIb (1) | | | | | |
|  | Crus II | left | -24.0 | -81.4 | -42.8 | 0.009 | 701 |
|  | Crus II | left | -34.2 | -78.0 | -41.1 | 0.01 | 693 |
|  | Crus I | left | -42.7 | -71.2 | -42.8 | 0.023 | 556 |
| 2 | Crus II (**10**) | left | -5.3 | -79.7 | -34.3 | 0.044 | 454 |
| *healthy controls > patients, early recall, CS- > rest* | | | | | | | |
|  | no surviving clusters | | | | | | |
| *healthy controls > patients, early recall, No-US post CS+ > rest* | | | | | | | |
|  | no surviving clusters | | | | | | |
| *healthy controls > patients, early recall, No-US post CS- > rest* | | | | | | | |
|  | no surviving clusters | | | | | | |
