# Supplementary material for: Mild Deficits in Fear Learning: Evidence from Humans and Mice with Cerebellar Cortical Degeneration
Source: eNeuro. 2024 Feb 22;11(2):ENEURO.0365-23.2023. doi: 10.1523/ENEURO.0365-23.2023 (PMC10897646; doi:10.1523/ENEURO.0365-23.2023)
Supplement: Table 12-1 — Results of the non-parametric two-way repeated measures ANOVA for freezing behavior between CT-shortPC and CT-longQ27PC mice groups. Download Table 12-1, DOC file. [file eneuro-11-ENEURO.0365-23.2023-s007.doc]

## Table 12-1. Results of the non-parametric two-way repeated measures ANOVA for freezing behavior between CT-short^PC^ and CT-longQ27^PC^ mice groups.

| ***n* CT-short^PC^*/ n* CT-longQ27^PC^** | **Factor** | **Num Df** | ***F*** | ***P*** |
| --- | --- | --- | --- | --- |
| **Pre-onset disease stage** | | | | |
| *fear acquisition training* | | | | |
| 10/10 | Genotype  Trial  Genotype × Trial | 1  3.37  3.37 | 0.35  52.72  1.15 | 0.554  **<.001*****  0.331 |
| *early extinction training* | | | | |
| 10/10 | Genotype  Trial  Genotype × Trial | 1  5.08  5.08 | 5.93  4.16  0.81 | **0.015***  **< .001*****  0.541 |
| *mid extinction training* | | | | |
| 10/10 | Genotype  Trial  Genotype × Trial | 1  5.4  5.4 | 1.75  0.94  0.84 | 0.186  0.455  0.532 |
| *late extinction training* | | | | |
| 10/10 | Genotype  Trial  Genotype × Trial | 1  5.59  5.59 | 0.58  1.25  0.99 | 0.448  0.279  0.425 |
| **Early disease stage** | | | | |
| *fear acquisition training* | | | | |
| 10/10 | Genotype  Trial  Genotype × Trial | 1  3.71  3.71 | 2.72  46.35  1.01 | 0.099  **<.001*****  0.397 |
| *early extinction training* | | | | |
| 10/10 | Genotype  Trial  Genotype × Trial | 1  5.13  5.13 | 8.18  3.43  1.27 | **0.004***  **0.004***  0.272 |
| *mid extinction training* | | | | |
| 9/10 | Genotype  Trial  Genotype × Trial | 1  5.09  5.09 | 1.05  4.66  0.97 | 0.301  **<.001*****  0.435 |
| *late extinction training* | | | | |
| 10/10 | Genotype  Trial  Genotype × Trial | 1  5.83  5.83 | 0.27  1.93  0.82 | 0.602  0.075  0.552 |
| **Late disease stage** | | | | |
| *fear acquisition training* | | | | |
| 15/15 | Genotype  Trial  Genotype × Trial | 1  4.01  4.01 | 1.32  91.75  0.67 | 0.250  **<.001*****  0.615 |
| *early extinction training* | | | | |
| 15/15 | Genotype  Trial  Genotype × Trial | 1  5.97  5.97 | 6.30  3.15  2.09 | **0.012***  **0.004***  0.052 |
| *mid extinction training* | | | | |
| 15/15 | Genotype  Trial  Genotype × Trial | 1  6.34  6.34 | 4.07  2.74  1.26 | **0.044***  **0.010***  0.272 |
| *late extinction training* | | | | |
| 15/15 | Genotype  Trial  Genotype × Trial | 1  5.90  5.90 | 1.79  1.33  0.35 | 0.181  0.241  0.908 |

* Significant results at *p* < 0.05.

*** Significant results at *p* < 0.001.
