# Supplementary material for: Mild Deficits in Fear Learning: Evidence from Humans and Mice with Cerebellar Cortical Degeneration
Source: eNeuro. 2024 Feb 22;11(2):ENEURO.0365-23.2023. doi: 10.1523/ENEURO.0365-23.2023 (PMC10897646; doi:10.1523/ENEURO.0365-23.2023)
Supplement: Table 12-2 — Results of the non-parametric two-way ANOVA-type statistic for freezing behavior between genotype and trial during baseline and retrieval. Download Table 12-2, DOC file. [file eneuro-11-ENEURO.0365-23.2023-s008.doc]

## Table 12-2. Results of the non-parametric two-way ANOVA-type statistic for freezing behavior between genotype and trial during baseline and retrieval.

| ***n* CT-short^PC^*/ n* CT-longQ27^PC^** | **Factor** | **Num Df** | ***F*** | ***P*** |
| --- | --- | --- | --- | --- |
| **Pre-onset disease stage** | | | | |
| 10/10 | Genotype  Trial  Genotype × Trial | 1  1  1 | 17.05  176.02  2.01 | **<.001*****  **<.001*****  0.156 |
| **Early disease stage** | | | | |
| 10/10 | Genotype  Trial  Genotype × Trial | 1  1  1 | 10.45  102.87  1.52 | **0.001***  **<.001*****  0.217 |
| **Late disease stage** | | | | |
| 10/10 | Genotype  Trial  Genotype × Trial | 1  1  1 | 0.84  56.80  0.224 | 0.360  **<.001*****  0.876 |

* Significant results at *p* < 0.05.

*** Significant results at *p* < 0.001.
