# Supplementary material for: Mild Deficits in Fear Learning: Evidence from Humans and Mice with Cerebellar Cortical Degeneration
Source: eNeuro. 2024 Feb 22;11(2):ENEURO.0365-23.2023. doi: 10.1523/ENEURO.0365-23.2023 (PMC10897646; doi:10.1523/ENEURO.0365-23.2023)
Supplement: Table 12-3 — Post hoc comparisons for freezing behavior between genotype and trial during baseline and retrieval (least squares means test). Download Table 12-3, DOC file. [file eneuro-11-ENEURO.0365-23.2023-s009.doc]

## Table 12-3. *Post hoc* comparisons for freezing behavior between genotype and trial during baseline and retrieval (least squares means test).

| **Genotype** | **Trial** | ***t*-value** | ***P*** |
| --- | --- | --- | --- |
| **Pre-onset disease stage** | | | |
| CT-short^PC^  CT-longQ27^PC^  CT-short^PC x^ CT-longQ27^PC^  CT-short^PC x^ CT-longQ27^PC^ | Baseline x Retrieval  Baseline x Retrieval  Baseline  Retrieval | *t*(18) = -8.79  *t*(18) = -10.79  *t*(18) = -2.39  *t*(18) = -4.04 | **< .001*****  **< .001*****  **0.028***  **< .001***** |
| **Early disease stage** | | | |
| CT-short^PC^  CT-longQ27^PC^  CT-short^PC x^ CT-longQ27^PC^  CT-short^PC x^ CT-longQ27^PC^ | Baseline x Retrieval  Baseline x Retrieval  Baseline  Retrieval | *t*(18) = -6.38  *t*(18) = -9.83  *t*(18) = -1.31  *t*(18) = -3.75 | **< .001*****  **< .001*****  0.206  **< .001***** |
| **Late disease stage** | | | |
| CT-short^PC^  CT-longQ27^PC^ | Baseline x Retrieval  Baseline x Retrieval | *t*(18) = -5.16  *t*(18) = -5.54 | **< .001*****  **< .001***** |

* Significant results at *p* < 0.05.

*** Significant results at *p* < 0.001
